# Interleukin Expression and Activation of Inflammatory/Apoptosis Pathways on Human Gingival Fibroblasts Exposed to Light-Cured Dental Composites

**DOI:** 10.3390/polym18202484

**Published:** 2026-10-11

**Authors:** Edoardo Sorrentino, Camillo D’Arcangelo, Antonella Mazzone, Luigia Fonticoli, Alessio Frisone, Ludovica Coppola, Emanuela Mazzon, Oriana Trubiani, Jacopo Pizzicannella, Guya Diletta Marconi, Francesca Diomede, Francesco De Angelis

**Affiliations:** 1 Department of Innovative Technologies in Medicine and Dentistry, University “G. d’Annunzio” Chieti-Pescara, Via dei Vestini 31, 66100 Chieti, Italy; sorrentinoed@libero.it (E.S.); antonella.mazzone@unich.it (A.M.); luigia.fonticoli@unich.it (L.F.); alessio.frisone@phd.unich.it (A.F.); emanuela.mazzon@unich.it (E.M.); oriana.trubiani@unich.it (O.T.); guya.marconi@unich.it (G.D.M.); 2 Department of Medical, Oral and Biotechnological Sciences, University “G. d’Annunzio” Chieti-Pescara, Via dei Vestini 31, 66100 Chieti, Italy; camillo.darcangelo@unich.it (C.D.); ludovicacoppola1@gmail.com (L.C.); fda580@gmail.com (F.D.A.); 3 Department of Neuroscience, Imaging and Clinical Sciences, University “G. d’Annunzio” Chieti-Pescara, Via dei Vestini 31, 66100 Chieti, Italy; jacopo.pizzicannella@unich.it

**Keywords:** composites, inflammation, apoptosis, polymerization

## Abstract

This in vitro study aimed to analyze the activation of the NFκB p65/MyD88/NALP3 inflammatory pathway and the expression of the BCL-2/BAX/Caspase-3 apoptosis pathway on human gingival fibroblasts (hGFs) after 24 h and 1-week exposure to the eluates of three light-cured commercial composites (GR: GrandioSO, VOCO; BF: Enamel Plus HRi Biofunction, Micerium; FU: Filtek universal restorative, 3M) and the production of pro-inflammatory cytokines (TNF-α, IL-6, IL-1β). A total of 50 light-cured disk-shaped specimens for each composite group (GR, BF and FU) were produced. The tested materials differed in resin matrix composition; notably, GR contained Bis-GMA, whereas BF and FU contained urethane-based dimethacrylates. The hGFs were exposed for 24 h and 1 week to eluates of each group. Pathway activation was evaluated by cell metabolic activity, immunofluorescence analysis and real-time PCR. Inflammatory effects were assessed in hGF culture by measuring proinflammatory cytokine production (ELISA). GR, BF and FU showed a reduction in metabolic activity in a time-dependent manner. GR showed a significant decrease in terms of cell viability after 1 week of exposure and a greater expression of proapoptotic markers than BF and FU. High levels of proinflammatory cytokines were measured after 1 week of culture for GR. The inflammatory pathway was activated in a time-dependent manner. Pathway activation was material-dependent; among the tested composites, GR was found to be more cytotoxic than BF and FU. Despite the limitations of an in vitro study, these findings suggest that dental composite eluates cause time- and material-dependent cytotoxicity, inflammation, and apoptosis in hGFs. Exposure significantly impairs cellular metabolic activity from 24 h up to 1 week. Among the evaluated materials, the GR composite triggered a markedly severe inflammatory and apoptotic effect compared to BF or FU. Ultimately, these findings underscore the potential clinical importance of selecting materials with optimal biocompatibility to minimize adverse tissue reactions, although their direct translation into clinical practice would require further research.

## 1. Introduction

The continuous demand for aesthetic and effective tooth-colored restorative materials led to the development and widespread diffusion of resin-based dental composites [1]. Composite resins are the most commonly used materials in modern restorative dentistry thanks to their minimally invasive application, excellent adhesive capabilities, and aesthetic and mechanical qualities [2,3,4]. Their wide-ranging versatility also makes them suitable materials for the cementation of dental prostheses [5]. Despite these advantages, challenges such as polymerization shrinkage, bacterial adhesion and cytotoxicity remain persistent concerns related to these materials [6,7,8]. Resin-based composites are made of an organic polymeric matrix containing fillers bonded thanks to a silane coupling agent: chemical agents are also included to promote or inhibit the polymerization reaction. Over time, doubts have been raised about their safe use [9,10,11,12,13] and their limits concerning biocompatibility [14,15]. Bisphenol-A-glycidyl-methacrylate (Bis-GMA) is the main monomer in the resin matrix and is composed by methyl methacrylate groups added to the hydroxyl groups of bisphenol A (BPA) via a glycidyl spacer [16]. It is generally mixed with other low-viscosity monomers such as bisphenol-A dimethacrylate (Bis-DMA), ethylene glycol-dimethyl acrylate (EGDMA), triethylene glycol dimethacrylate (Teg-DMA), methyl-methacrylate (MMA) or urethane dimethacrylate (UDMA) [17,18]. Bis-GMA provides an appropriate degree of viscosity, easing manufacturing processes and clinical handling [19]. It also helps decrease volumetric shrinkage following polymerization reaction and provides adequate mechanical properties, high refractive index and reduced volatility [20,21]. Nevertheless, its widespread employment should be carefully evaluated, as it has been shown that Bis-GMA and other bisphenol A (BPA)-derived monomers can promote cytotoxicity and genotoxicity through release/inhibition of cytokines and DNA damage [22,23]. However, it has recently been shown how the whole formulation, rather than the presence/absence of individual monomers, can better affect the overall biocompatibility [7].

Curing reaction is defined as the degree of conversion of monomers into a polymer network. Previous studies have shown that the polymerization process results in lower concentrations of residual monomer, although it never becomes completely absent, leading to a potentially prolonged contact with the oral mucosa, whose biological effect on human gingival fibroblasts must seemingly be evaluated [24]. In clinical practice, resin-based composites are often polymerized chairside for 20–40 s using dedicated light-curing units [25]. However, following such a direct approach, a high percentage of material polymerization is not guaranteed [26] and it has been found that up to 45% of unreacted monomers may remain unpolymerized [27]. Indirect restorations can be subjected to an additional heat-curing step before adhesive cementation [3,5,25]. Heat-curing protocol promotes a higher, though still not complete, degree of conversion [28,29], thereby improving properties such as microhardness, flexural strength, fracture toughness, wear resistance, tensile strength, and color stability [30,31,32,33]. The simultaneous enhancement of mechanical properties as a result of the increase in monomeric conversion should reduce the amount of unreacted material at the composite surface [34]. As heat-cured resin-based composites have already been tested, the innovation lies in subjecting light-cured composites to cytotoxic pathways similar to those previously tested for heat-cured ones, given that light-cured resins are used very frequently in clinical practice [35]. Uncured monomers can be externally released, possibly leading to allergic reactions, irritation and a proinflammatory response [13,36]. They have been found to significantly reduce cellular viability, leading to cytotoxic effects. Therefore, it is essential to investigate the impact of direct restorations due to the possible high number of uncured monomers [27], particularly regarding potential consequences for biocompatibility [37].

From a clinical point of view, gingival inflammation and recession have been reported in cases of closeness or direct contact between composite restorations and the periodontium [38]. Also, when composites’ monomeric elution and diffusion spread through dentinal tubules, pulp reactions can be observed, potentially jeopardizing pulp vitality [39].

The inflammatory pathway NFκB p65/MyD88/NALP3 is involved in inflammation and in the innate and adaptive immune system [40,41]. NALP3 inflammasome is activated by its components’ overexpression, promoted by damage-associated molecular patterns (DAMPs) or pathogen-associated molecular patterns (PAMPs), through specific receptors such as TLRs. Also, NFκB can modulate the activation of NALP3, working as a transcription factor [42].

In the oral cavity, human gingival fibroblasts (hGFs) act as primary immunomodulatory sentinels rather than purely passive structural elements. Within these fibroblasts, the NALP3 inflammasome serves as a vital intracellular danger sensor. When hGFs are exposed to xenobiotic stressors like dental resin eluates, these chemical components act as intracellular DAMPs that accelerate this pathway, positioning the fibroblasts as active drivers of the localized periodontal inflammatory cascade [43].

Cytotoxicity resulting from the disruption of DNA is related to apoptosis [44]. Apoptosis is regulated by different factors such as B cell lymphoma 2 (BCL-2), BAX, and Caspase-3 (CASP-3) [45]. The antiapoptotic BCL-2 protein either activates or modulates the intrinsic apoptosis pathways [46]. BAX is a proapoptotic protein involved in regulating the intrinsic apoptosis pathway triggered by the induction of cytochrome C release [47]. The proapoptotic CASP3 protein triggers DNA breakage and fragmentation [44,48,49]. Physiologically, material-induced cellular stress shifts the delicate homeostatic balance within the hGF mitochondrial apoptosis pathway. Instead of maintaining survival through BCL-2, severe chemical exposure causes proapoptotic BAX to induce mitochondrial outer membrane permeabilization. This triggers the release of cytochrome C into the hGF cytoplasm, resulting in the activation of executioner CASP-3, which drives the final programmed cell death of the gingival tissue [50,51].

Inflammatory processes, as well as diseases, disability and mortality in the elderly, have been also strongly correlated to high levels of tumor necrosis factor (TNF), interleukin-6 (IL-6) and Interleukin-1β (IL-1β) [52,53]. Specifically, TNF-α is a proinflammatory cytokine producing inflammatory molecules, chemokines and cytokines, exhibiting pleiotropic effects on multiple cell types; it is involved in the pathogenesis of various chronic inflammatory diseases [54,55,56]. IL-6 is a proinflammatory cytokine that stimulates local synovial leukocytes’ activation and the production of antibodies [57,58]: it is secreted by leukocytes and stromal cells for a maximum of 15 h at local inflammatory sites [59]. Finally, IL-1β is a proinflammatory cytokine involved in host defense responses to infection and injury [60]. It is produced as an inactive protein called pro-IL-1β following recognition of molecules carried by pathogens known as “pathogen-associated molecular patterns” (PAMPs) [61].

Expanding the current knowledge about inflammatory and apoptotic pathways triggered on cells following their exposure to dental materials may represent a first and paramount step towards possible biocompatibility optimization. As a consequence, the aim of the present study was to evaluate the activation of the NFκB p65/MyD88/NALP3 inflammatory pathway and the BCL-2/BAX/CASP-3 apoptosis pathway expression on human gingival fibroblasts (hGFs) after 24 h and 1-week exposure to the eluates of three already marketed light-cured dental composites. The inflammatory effects were also analyzed by measuring the production of proinflammatory cytokines (TNF-α, IL-6, IL-1β). The null hypothesis tested was that no differences in the pathway activation and in the inflammatory effects could be observed when comparing the three composite resin eluates to one another or when comparing them to a control group, which included hGFs not exposed to any composite eluate.

## 2. Materials and Methods

A list of the tested materials is shown in Table 1.

### 2.1. Composite Disk Manufacture and Eluate Preparation

To produce disk-shaped specimens (diameter, 4 mm; height, 2 mm; total surface area, 50.27 mm^2^), uncured composites were placed in a polyvinylsiloxane mold between two glass slides stuck together with a paper clip, which allows one to remove surplus material and obtain smooth and standardized surfaces. A 20 s light-curing cycle was performed on each specimen, from top surface, employing a light-emitting diode curing unit (Celalux 3, VOCO, Cuxhaven, Germany) with 8 mm diameter tip and an output power of 1300 mW/cm^2^. This protocol was consistent with the manufacturers’ instructions for use for GR, BF and FU, which recommend a maximum increment thickness of 2 mm and a 20 s curing time [62,63,64]. A total of 50 specimens were manufactured with each different material under investigation.

The eluates were prepared in accordance with ISO 10993-12 [65]. Specifically, Dulbecco’s Modified Eagle’s Medium (DMEM, Lonza, Walkersville, MD, USA) was added at a standard surface area-to-volume ratio of 3 cm^2^/mL of disks previously prepared. The medium was kept in contact with the resins for 72 h at 37 °C. Once collected, the eluates were used immediately [66].

### 2.2. Cell Culture Establishment

hGFs (PCS-201-018 ATCC, Manassas, VT, USA) were cultured in Fibroblast Basal Medium (ATCC PCS-201-030) in addition to Fibroblast Growth Kit-Low Serum (ATCC PCS-201-041), containing 5 ng/mL of rh FGF b, 7.5 mM of L-glutamine, 50 μg/mL ascorbic acid, 1 μg/mL of Hydrocortisone Hemisuccinate, 5 μg/mL of rh insulin and 2% fetal bovine serum. The culture was maintained in an incubator at 37 °C in a humidified atmosphere with 5% CO_2_ and 95% air, and when the cells reached 75–80% confluence, subcultures were produced [67]. All the experiments were performed with cells processed between 4 and 8 passages, and each assay was performed in triplicate.

### 2.3. Cell Metabolic Activity (MTS Assay)

The metabolic activity of hGFs cultured alone (control group, CTRL) or with eluates derived from the three tested composites was analyzed with the 3-(4,5-dimethylthiazol-2-yl)-5-(3-carboxymethoxyphenyl)-2-(4-sulfo-phenyl)-2H-tetrazolium (MTS) assay (CellTiter 96^®^ Aqueous One Solution Cell Proliferation Assay, Promega, Madison, WI, USA). For the MTS assay, 2.4 × 10^3^ hGFs/well were seeded into 96-well plates and maintained in culture at 37 °C with DMEM (Lonza) supplemented with 10% of FBS (Lonza) for 24 h, 48 h, 72 h and 1 week. Subsequently, 20 μL/well of MTS staining solution was added and the plates were incubated at 37 °C for 3 h. Formazan salts quantification was analyzed through 490 nm wavelength absorbance with Synergy™ HT Multi-Detection Microplate Reader (Biotech, Winooski, VT, USA). MTS assay was executed in triplicate [68].

### 2.4. Experimental Study Design

hGFs were cultured with and without the eluates derived from GR, BF and FU for 24 h and 1 week, according to the following scheme:

- hGFs cultured with complete medium for 24 h;
- hGFs cultured with eluate derived from GR for 24 h;
- hGFs cultured with eluate derived from BF for 24 h;
- hGFs cultured with eluate derived from FU for 24 h;
- hGFs cultured with complete medium for 1 week;
- hGFs cultured with eluate derived from GR for 1 week;
- hGFs cultured with eluate derived from BF for 1 week;
- hGFs cultured with eluate derived from FU for 1 week.

The experimental study design was performed in triplicate.

### 2.5. Confocal Laser Scanning Microscope (CLSM)

hGFs cultured with eluates (test groups) and with complete medium (CTRL groups) were seeded in 8-well culture slides (Corning, Glendale, AZ, USA) (density of 6.4 × 10^4^/well); then, after 24 h and 1 week, they were fixated with 0.4% paraformaldehyde (PFA) (BioOptica, Milan, Italy) for 45 min at room temperature. Samples were washed three times in PBS and permeabilized for 6 min in 0.1% Triton X-100 (BioOptica), blocked for 1 h with 5% non-fat milk in PBS and incubated overnight at 4 °C with the primary antibodies anti-NFκB p65 (sc-8008, Santa Cruz Biotechnology, Dallas, TX, USA), anti-MyD88 (sc-136970, Santa Cruz Biotechnology), anti-NALP3 (sc-134306, Santa Cruz Biotechnology), anti-BCL-2 (sc-7382, Santa Cruz Biotechnology), anti-BAX (sc-493, Santa Cruz Biotechnology) and anti-CASP-3 (sc-56052, Santa Cruz Biotechnology) at the concentration of 1:200. All samples were first incubated with the secondary antibodies Goat anti-Mouse IgG (H + L) Highly Cross-Adsorbed Secondary Antibody, Alexa Fluor 568 (A11031, Invitrogen, Thermo Fisher Scientific, Waltham, MA, USA) and Goat anti-Rabbit IgG (H + L) Cross-Adsorbed Secondary Antibody, Alexa Fluor 568 (A11011, Invitrogen) at the concentration of 1:200 for 1 h at 37 °C and then with Alexa Fluor 488 phalloidin green fluorescent conjugate (A12379, Invitrogen) and TOPRO (T3605, Invitrogen) to stain, respectively, actin and nuclei. Immunofluorescence was evaluated with image acquisition using the Zeiss LSM800 confocal system (Carl Zeiss, Jena, Germany) equipped with a Plan Neofluar oil-immersion objective (40 × 1.3 NA). Images were collected using an argon laser beam with excitation lines at 488 nm and a helium–neon source (543 and 665 nm) with an image size resolution of 1024 × 1024 pixels [69].

### 2.6. RNA Isolation and Real-Time RT-PCR Analysis

PureLink RNA Mini Kit (Ambion, Thermo Fisher Scientific, Milan, Italy) was employed to extract total RNA of all the samples. Once RNA was obtained, the retro transcription of each sample was realized using M-MLV Reverse Transcriptase (M1302 Sigma-Aldrich, Saint Louis, MO, USA). To obtain 50 µg of cDNA, 1 µg of total RNA was employed, as recommended in the technical bulletin. Then, the Mastercycler ep real plex real-time PCR system (Eppendorf, Hamburg, Germany) was used to produce a real-time PCR. The cDNA from the samples was amplificated following the steps below (3 min at 95 °C, 40 cycles of 15 s at 95 °C, and 1 min at 60 °C) in order to evaluate the gene expression of *RELA* (Hs.PT.58.22880470 IDT), *MYD88* (Hs.PT.58.40428647.g), *NALP3* (Hs.PT.58.39303321), *BCL2* (Hs.PT.56a.2905156), *BAX* (Hs.PT.56a.19141193) and *CASP3* (Hs.PT.56a.25882379.g). *B2M* (Hs.PT.58v.18759587) expression was evaluated in all the samples as housekeeping gene. All the primers were purchased from Integrated DNA Technologies (IDT, Tema Ricerca, Bologna, Italy) (Table 2). The expression levels for each gene were obtained through the 2^−ΔΔCt^ method. Real-time PCR was executed in three independent experiments [70].

### 2.7. ELISA Assay

The levels of TNF-α, IL-6 and IL-1β were measured using a commercial ELISA kit (SKFI0014, Assay Genie, Dublin, Ireland) according to the manufacturer’s guidelines. In brief, 100 µL of standards or samples were added to each well and incubated for 2 h at room temperature. Wells were then washed 3 times with the provided wash buffer and incubated with 100 µL of detection antibody for 1 h. After washing, 100 µL of substrate solution was added, and the reaction was allowed to proceed for 15 min in the dark. The reaction was stopped by adding 50 µL of stop solution, and absorbance was measured at 450 nm using a microplate reader (Synergy™ HT Multi-Detection Microplate Reader, Biotech). Each sample was assayed in triplicate, and concentrations were calculated based on the standard curve calculated with GraphPad software (version 10.1.1).

### 2.8. Statistical Analysis

The statistical analysis was carried out using one-way ANOVA and post hoc Tukey’s multiple comparisons test through GraphPad 5 (v. 5.04, GraphPad, San Diego, CA, USA) software. Differences were considered statistically significant for values of *p* < 0.05. Gene expression data after real-time PCR was obtained with comparative 2^−ΔΔCt^ method.

## 3. Results

### 3.1. Cell Viability in hGFs

After the hGFs’ exposition to GR, BF and FU eluates for 24 h, 48 h, 72 h and 1 week of culture, a reduction in the cell viability was detected in all samples (Figure 1). Specifically, GR showed a slight decrease in the cell viability after 24 h of exposure when compared to the CTRL group, while a significant decrease in cell viability was observed after hGFs’ exposure to GR, BF and FU eluates after 72 h and 1 week compared to the CTRL group. After 72 h of exposure, a decrease in cell viability was evidenced after exposure to GR eluate compared to BF and FU eluates, which became more significant after 1 week of exposure.

### 3.2. Expression Levels of NFκB p65/MyD88/NALP3 in hGFs Exposed to GR, BF, and FU for 24 H and 1 Week

The proinflammatory markers NFkB p65, MyD88 and NALP3 after 24 h of treatment evidenced an immunofluorescence upregulation of the marker NFκB p65 after hGFs exposure to GR and FU eluates in hGF culture (Figure 2). An upregulation of NALP3 was also detected with GR eluate when compared to the CTRL group (Figure 3), while no evidence of MyD88 over- or under-regulation was observed (Figure 4).

Immunofluorescence images of the inflammatory marker expressions after 1 week of exposure showed an upregulation of the level of inflammatory proteins compared to the CTRL group (Figure 5, Figure 6 and Figure 7). Specifically, GR eluate in hGF culture showed a significant up-regulation of NFkB p65, MyD88 and NALP3 compared to treatment with BF and FU eluates (Figure 5, Figure 6 and Figure 7).

Immunofluorescence data were validated by immunofluorescence quantitative analysis expression (Figure 8 and Figure 9).

### 3.3. Expression Levels of BCL-2/BAX and CASP-3 in hGFs Exposed to GR, BF, and FU for 24 H and 1 Week

Immunofluorescence expression of the apoptotic markers after 24 h of treatment showed a BCL-2 decrease in the CTRL group and GR eluate compared to BF and FU exposure (Figure 10). On the other hand, the level of BAX and CASP-3 showed a slight increase after GR treatment compared to hGFs (Figure 11 and Figure 12).

After 1 week of culture, apoptotic markers expression showed a significant upregulation when compared to the CTRL group (Figure 13, Figure 14 and Figure 15). BCL-2 expression decreased in hGFs treated with GR compared to BF and FU (Figure 13). hGFs treated with BF eluate showed a significant BCL-2 expression increase in comparison with the CTRL group and to GR and FU (Figure 13). On the other hand, the level of proapoptotic BAX and CASP-3 markers increased after GR exposure when compared to BF and FU eluates (Figure 14 and Figure 15).

Immunofluorescence data were validated by immunofluorescence quantitative analysis expression (Figure 8 and Figure 9).

### 3.4. Expression Levels of Inflammatory and Apoptotic Markers in hGFs by RT-PCR Analysis After 24 H and 1 Week of Exposure

The results obtained by confocal microscopy and immunofluorescence were confirmed by RT-PCR analysis (Figure 16 and Figure 17).

In detail, RT-PCR analysis regarding *RELA*, *MYD88*, *NALP3*, *BCL2*, *BAX* and *CASP3* expression confirmed the results evidenced by confocal microscopy (Figure 16 and Figure 17), showing an upregulated expression of *RELA*, *MYD88* and *NALP3* after 1-week exposure after treatment with GR, BF and FU eluates when compared to the CTRL group (Figure 16). A downregulation of *BCL2* with GR eluate was detected after 1 week of culture when compared to the other groups and an upregulation of *BAX* and *CASP3* expression after treatment with GR when compared to BF and FU was detected after 1 week of culture (Figure 17).

### 3.5. Evaluation of Proinflammatory Cytokine Production by ELISA Assay

To evaluate the inflammatory activity of GR, BF and FU on hGFs, the proinflammatory cytokine production (TNF-α, IL-6 and IL-1β) was measured. Results are expressed as a concentration (pg/mL) relative to the untreated cells (hGFs), as shown in Figure 18.

A high concentration of TNF-α was measured in GR-exposed cells after both 24 h and 1 week of treatment. On the other hand, IL-6 and IL-1β appeared modulated only after 1 week of culture on GR-exposed cells when compared to the untreated cells and to the BF- and FU-exposed ones.

## 4. Discussion

The cytotoxic effects promoted by the same time points (24 h, 1 week) and the same resin-based composites (GR, BF, FU) investigated in the present research had been already analyzed in a previous study [71] but focusing on the heat-cured materials, which simulates an indirect restorative approach. Clinically, many small cavity restorations are placed following a direct protocol and are light-cured chairside, which precludes any chance for a deeper degree of polymerization. Since light-cured direct composites, compared to heat-cured ones, have higher amounts of unpolymerized monomers [34], that might be more easily eluted in the culture medium, as well as in saliva [1], it seemed interesting to further analyze the specific cytotoxic behavior and the inflammatory response promoted by the light-cured materials [13,36,71]. In clinical dental practice, direct composite restorations are light-cured chairside for 20–40 s [25]. Since a 20 s curing time has been shown to produce a cytotoxicity rate like that of a 40 s curing time [72], a light-curing protocol of 20 s on one single side was adopted because resin composites are clinically polymerized on a single surface in such a way as to better reproduce in this in vitro study the monomer release that generally occurs in in vivo polymerization. Although this protocol reflects a clinically relevant curing condition, single-sided irradiation may result in a lower degree of conversion at the surface opposite to the light source and consequently in greater residual monomer release, which should be considered when interpreting the present findings. GR, BF and FU have a different chemical composition (Table 1). Among the tested composites, the monomer Bis-GMA is only included in GR formulation, suggesting this as a potential hazard for the more pronounced cytotoxicity herein observed. The cytotoxic potential of Bis-GMA is closely linked to the presence of Bisphenol A (BPA) derivative in its formulation [36,73,74], which can lead to proinflammatory cytokine production [75] and seems to be involved in the pathogenesis of various disorders [76]. A composite able to reduce or even eliminate the inflammatory pathways’ activations on hGFs should be positively considered by the dental practitioners, while an overstimulation of the same pathways could determine adverse or allergic reactions. Clinically this might become potentially detrimental for periodontal tissues [77,78,79], exacerbating any risk for gingival inflammation, recession or loss of attachment connected with the presence of iuxta-gingival or intrasulcular tooth restoration margins [38,80]. Biological width acts as a protective barrier against bacterial invasion and promotes periodontal stability [81]. Violation of the biological width by dental resins may trigger inflammation through both physical impingement and chemical toxicity from their eluates. To preserve the integrity of the supracrestal tissue attachment, restorative materials must demonstrate biocompatibility and should ideally promote cellular adhesion of the gingival epithelium. It is well known that the eluates released from resin-based dental materials, such as the three commercial composites herein investigated (GR, BF, and FU), can lead to inflammation, which may negatively impact the health and the integrity of the biological width [82]. Thus, in the present study, the specific inflammatory and apoptotic pathways activated in hGFs following exposure to those resin eluates have been better analyzed. It is worth noting that the findings of in vitro studies must be carefully interpreted, as they may differ from in vivo performance.

Although not directly examined in this study, from a clinical point of view, it could be assumed, with due caution, that cytotoxic monomers released through the dentinal tubules could also cause adverse reactions in the pulp, ranging from reversible to irreversible pulpitis and pulp necrosis, ultimately jeopardizing pulp vitality and long-term tooth prognosis [39].

The current work aimed to evaluate and compare the biological effects of GR, BF and FU eluates at different time points in an in vitro model on hGFs. The null hypothesis tested had to be rejected. Differences regarding pathways activation were detected when comparing the three light-cured composite resin eluates to one another or when comparing them to the control group.

While many pathways regulate generic cellular stress responses, the NFκB/MyD88/NALP3 axis represents the definitive, sequence-specific cascade governing both the initial priming and the downstream execution of the innate immune response within periodontal tissues. MyD88 and NFκB p65 drive the transcription of NALP3 components, while the assembled NALP3 inflammasome converts pro-IL-1β into its active, tissue-destructive form [42].

Crucially, this inflammatory cascade directly couples with the BCL-2/BAX/Caspase-3 intrinsic apoptotic pathway. Resin monomer exposure triggers severe cellular stress; when proapoptotic BAX overpowers BCL-2, it drives mitochondrial outer membrane permeabilization (MOMP). This structural failure activates executioner Caspase-3 to initiate programmed cell death while concurrently leaking mitochondrial danger signals into the cytoplasm, providing the vital secondary stimulus required for NALP3 inflammasome assembly [83].

Based on this knowledge, the inflammatory event was studied by analyzing the modulation of the NFκB p65/MyD88/NALP3 inflammatory pathway and the expression of the BCL-2/BAX/Caspase-3 pathway to evaluate the biological effects of free monomers on the resins’ surfaces.

The NFκB p65/MyD88/NALP3 pathway is a critical signaling axis in the innate immune response that links the detection of danger to the release of powerful inflammatory cytokines.

The MyD88-dependent signaling cascade initiates when specialized cell receptors detect pathogens or damage, triggering the MyD88 adapter protein to activate the NF-κB p65 subunit, which then translocates into the nucleus to drive the transcription of NALP3 inflammasome components and proinflammatory precursors like pro-IL-1β, effectively priming the cell for a second activation signal that assembles the inflammasome complex to release mature, active cytokines [84].

The BCL-2/BAX/Caspase-3 axis defines the intrinsic apoptotic pathway wherein the ratio between the antiapoptotic protein BCL-2 and the proapoptotic protein BAX determines mitochondrial membrane permeability; specifically, when BAX outweighs BCL-2, it triggers the release of cytochrome c, which ultimately activates Caspase-3 to execute the irreversible proteolytic cleavage of cellular structures [85].

Our in vitro study reported that after 1 week, hGF cells cultured with eluates derived from GR, BF and FU resins evidenced an upregulation in the expression of inflammatory mediators, such as NFκB p65, MyD88, and NALP3, compared to the untreated cells; meanwhile, hGF cells cultured with eluate derived by GR evidenced a significant expression of inflammatory markers compared to BF and FU resins as established by immunofluorescence analysis. In addition, after 24 h of culture, a decrease in the antiapoptotic marker BCL-2 after treatment with GR eluates was detected compared to CTRL, BF and FU, and an increase in proapoptotic CASP-3 was observed after GR exposition compared to the CTRL group. Following 1 week of culture, the antiapoptotic BCL-2 marker expression decreased in hGFs treated with GR when compared to BF and FU exposure, while the level of the proapoptotic BAX and CASP-3 markers increased after GR exposure when compared to BF and FU eluates. However, both GR, BF and FU showed an increase in BAX and CASP-3 markers.

In parallel, the cells cultured with eluate derived by GR, after 72 h and 1 week of culture, reported a significant decrease in terms of cell viability compared to the cells cultured with BF and FU as recognized by MTS analysis.

The genes’ expression of *NFκB p65/MyD88/NALP3* and *BCL-2/BAX/CASP-3* was also evaluated and confirmed the results obtained in confocal microscopy. A significant gene expression of the inflammation and apoptosis pathways was observed to be time-dependent, with a more pronounced expression after 1 week than after 24 h of exposure to eluates.

Unlike previous research [71], this study includes an ELISA test. It is one of the most specific and straightforward methods to detect biomolecules in both research and clinical settings. To further support the result obtained, we also evaluate cytokines such as TNF-α, IL-6, and IL-1β through an ELISA assay. In detail, the ELISA assay showed high TNF-α concentration in GR samples after 24 h and 1 week of culture, while the proinflammatory cytokine IL-6 was slightly higher after 1 week of GR culture when compared to CTRL and BF. Finally, GR showed a higher IL-1β expression after 1 week of culture than CTRL, BF and FU. Among the tested composites, GR seemed to be more exposed to proinflammatory cytokines than BF and FU.

Within the limitations of this in vitro study, GR showed a greater cytotoxicity among the tested composites. Nevertheless, some mediator alterations were also observed following BF and FU exposure: as a matter of fact, all composites may potentially cause, to a certain extent, an inflammation process, and the specific composite composition definitely plays a key role in their ultimate biocompatibility [71]. These findings are consistent with those of De Angelis and coll. [71], who reported that the activation of inflammatory and apoptotic pathways was material-dependent, being less evident with BF and FU, which further supports the key role of the specific material composition in the biological response of hGFs’ culture. This is also in agreement with previous evidence showing high levels of Teg-DMA release by GR after 24 h of soaking in culture medium [7]. Conversely, increased activation of the NF-κB p65/NLRP3/IL-1β inflammatory pathway was observed after hGFs’ adhesion to temporary dental resin containing Bis-GMA [86]. This suggests that both resin formulation and the type of exposure (direct contact or contact with eluate) may affect the biocompatibility of the resin-based materials. Nevertheless, the in vitro results of the present study cannot be directly extrapolated to clinical dental practice, and further studies are needed.

Dental composite cytotoxicity has been the subject of many previous studies: many of them focused just on the effects of each specific material component [87,88,89]. In vitro studies employing composites eluates allow one to analyze the effects of all components in dilution, which, in our opinion, mimic more closely the cell exposure occurring in the oral cavity than single-component experiments [90].

The strength of the present study lies in the fact that it examined not only the changes in the metabolic activity taking place during dental composites’ exposure but also the inflammatory and apoptosis pathways and their biological mechanism. Nevertheless, care should be taken when applying these in vitro results to clinical dental practice; in the oral cavity, a continuous wash-out action of the compounds released by dental polymers takes place [91], and the oral mucosa is much more resistant to cytotoxic agents than cell cultures. Further studies should expand these in vitro findings by adding analysis of other molecular mechanisms potentially involved in the inflammatory and apoptosis pathways and possibly investigate how the medium used to collect the eluate and the curing time may affect the release of uncured monomers. Furthermore, additional studies may be carried out to analyze more or other time points and resin-based materials to expand knowledge for a potential, more informed use of composites on a larger scale. Moreover, variations in the chemical composition of resins, polymerization times, and postprocessing methods across different manufacturers and technologies make it challenging to generalize the present findings to the huge number of restorative dental composites currently available. Future research should include a wider range of brands and formulations to gain a deeper and more comprehensive understanding of material variability and performance.

## 5. Conclusions

Within the limitations of an in vitro study, it can be concluded that the activation of inflammatory and apoptotic pathways following the exposure of hGFs to dental resin composite eluates was time-dependent. A marked reduction in the metabolic activity after 24 h and 1 week of exposure was detected. Also, cytotoxicity was material-dependent; among all tested composites, GR showed a greater activation of inflammatory and apoptotic pathways and higher inflammatory effects on hGFs than BF and FU. These findings may have potential implications for the clinical selection of resin-based dental composites with favorable biocompatibility, although their direct translation into clinical practice would require further research.

## Figures and Tables

**Figure 1:**
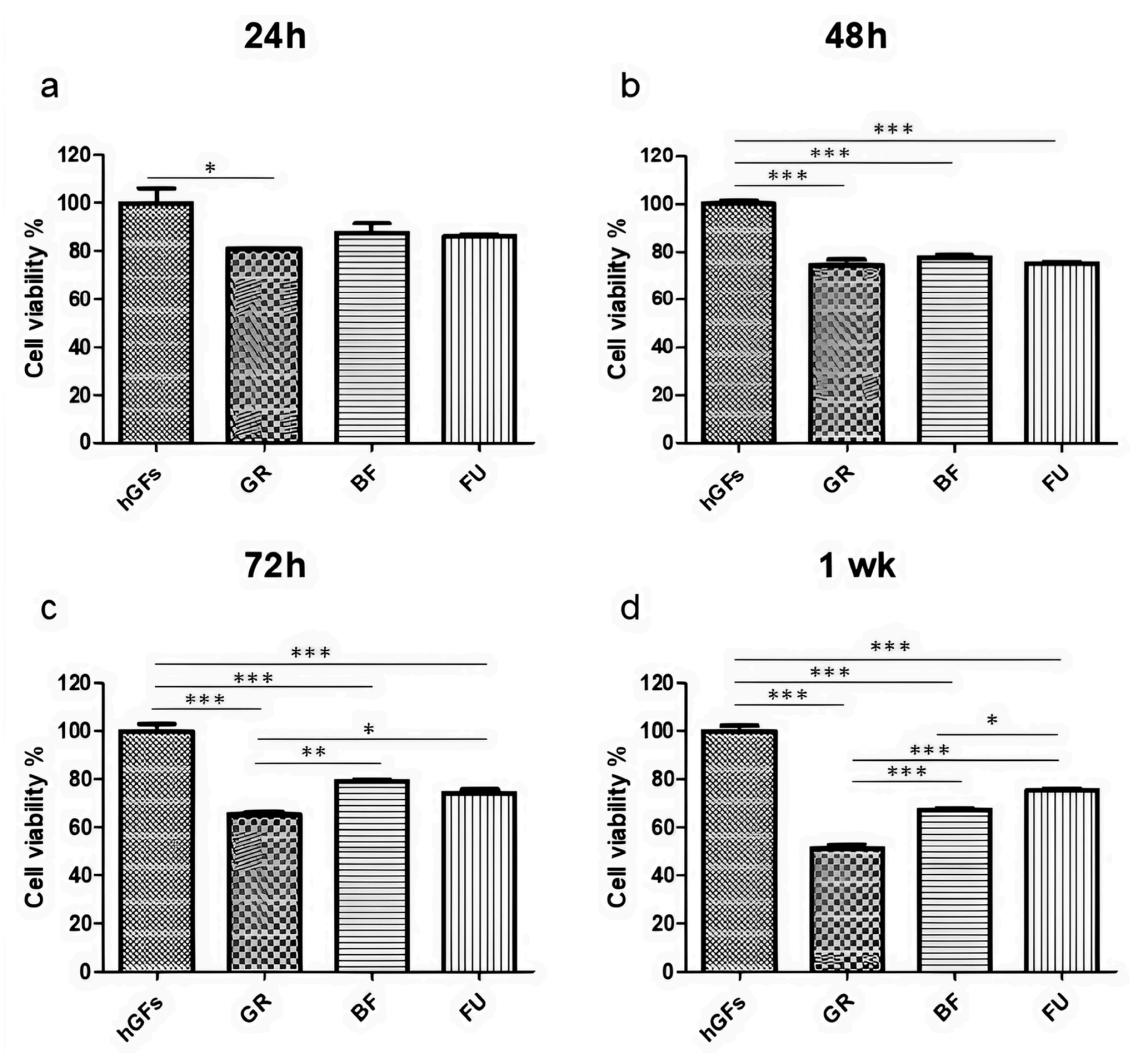
Cell metabolic activity (MTS). Effect of GR, BF, and FU on hGFs after 24 h, 48 h, 72 h, and 1 week of treatment. (**a**) Cell viability analyzed at 24 h of culture. (**b**) Cell viability analyzed at 48 h of culture. (**c**) Cell viability analyzed at 72 h of culture. (**d**) Cell viability analyzed after 1 week of culture. Results are shown as mean ± standard error (SEM) (*n* ≥ 3) of the optical percentage (%) of metabolically active cells (* *p* < 0.05, ** *p* < 0.01, *** *p* < 0.001).

**Figure 2:**
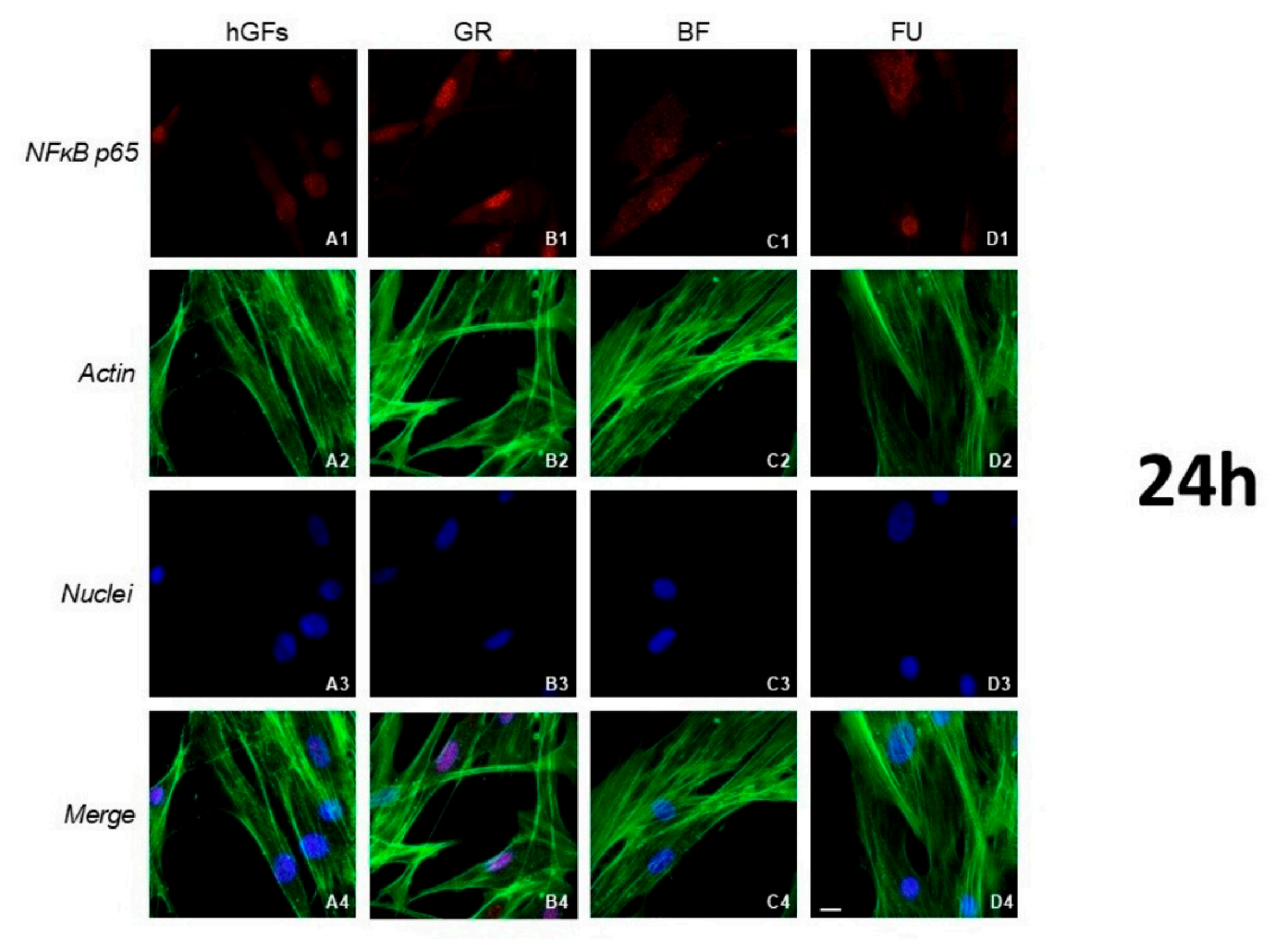
Immunofluorescence detection of NFkB p65 in hGFs (**A1**) and after 24 h of exposure to GR (**B1**), BF (**C1**) and FU (**D1**) (red channel); actin image of all channels in the CTRL group (**A2**) and after 24 h exposure to GR (**B2**), BF (**C2**) and FU (**D2**); nuclei images of all channels in the CTRL group (**A3**) and after 24 h exposure to GR (**B3**), BF (**C3**) and FU (**D3**). Merged image of all channels (**A4**) and 24 h exposure to GR (**B4**), BF (**C4**) and FU (**D4**). Green fluorescence: cytoskeleton actin. Red fluorescence: NFkB p65. Blue fluorescence: cell nuclei. Mag: 40×. Scale bar: 10 µm.

**Figure 3:**
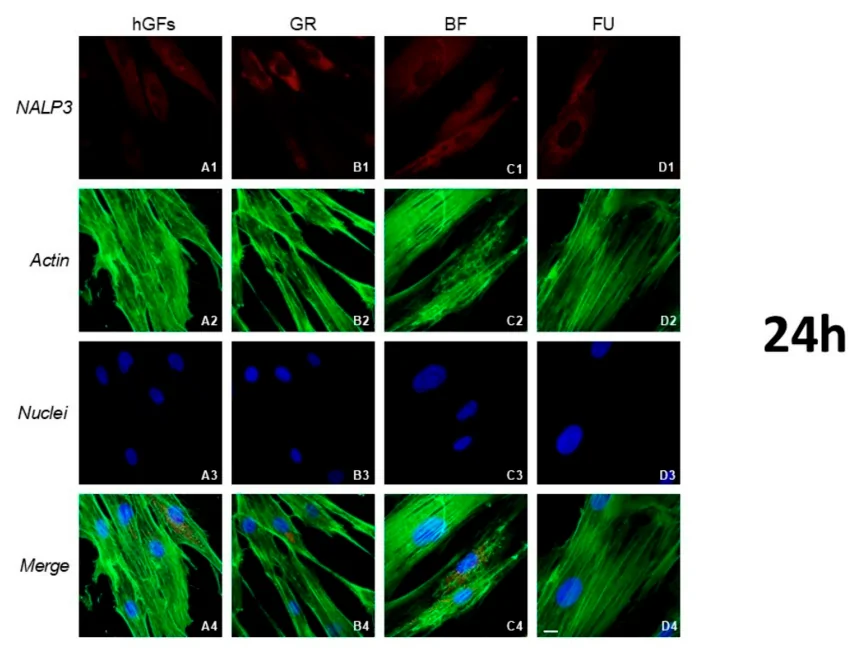
Immunofluorescence detection of NALP3 in hGFs (**A1**) and after 24 h of exposure to GR (**B1**), BF (**C1**) and FU (**D1**) (red channel); actin image of all channels in the CTRL group (**A2**) and after 24 h exposure to GR (**B2**), BF (**C2**) and FU (**D2**); nuclei images of all channels in the CTRL group (**A3**) and after 24 h exposure to GR (**B3**), BF (**C3**) and FU (**D3**). Merged image of all channels (**A4**) and 24 h exposure to GR (**B4**), BF (**C4**) and FU (**D4**). Green fluorescence: cytoskeleton actin. Red fluorescence: NALP3. Blue fluorescence: cell nuclei. Mag: 40×. Scale bar: 10 µm.

**Figure 4:**
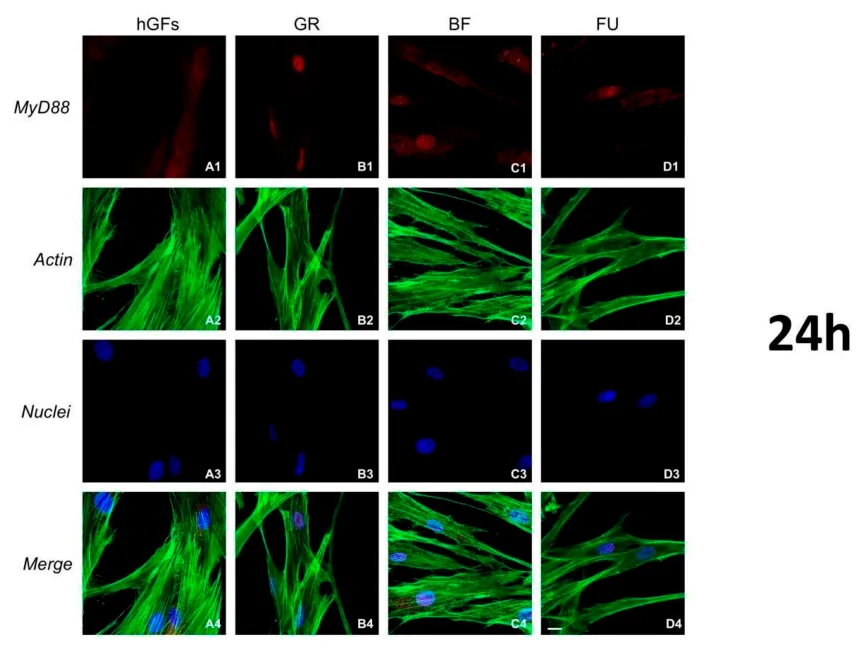
Immunofluorescence detection of MyD88 in hGFs (**A1**) and after 24 h of exposure to GR (**B1**), BF (**C1**) and FU (**D1**) (red channel); actin image of all channels in the CTRL group (**A2**) and after 24 h exposure to GR (**B2**), BF (**C2**) and FU (**D2**); nuclei images of all channels in the CTRL group (**A3**) and after 24 h exposure to GR (**B3**), BF (**C3**) and FU (**D3**). Merged image of all channels (**A4**) and 24 h exposure to GR (**B4**), BF (**C4**) and FU (**D4**). Green fluorescence: cytoskeleton actin. Red fluorescence: MyD88. Blue fluorescence: cell nuclei. Mag: 40×. Scale bar: 10 µm.

**Figure 5:**
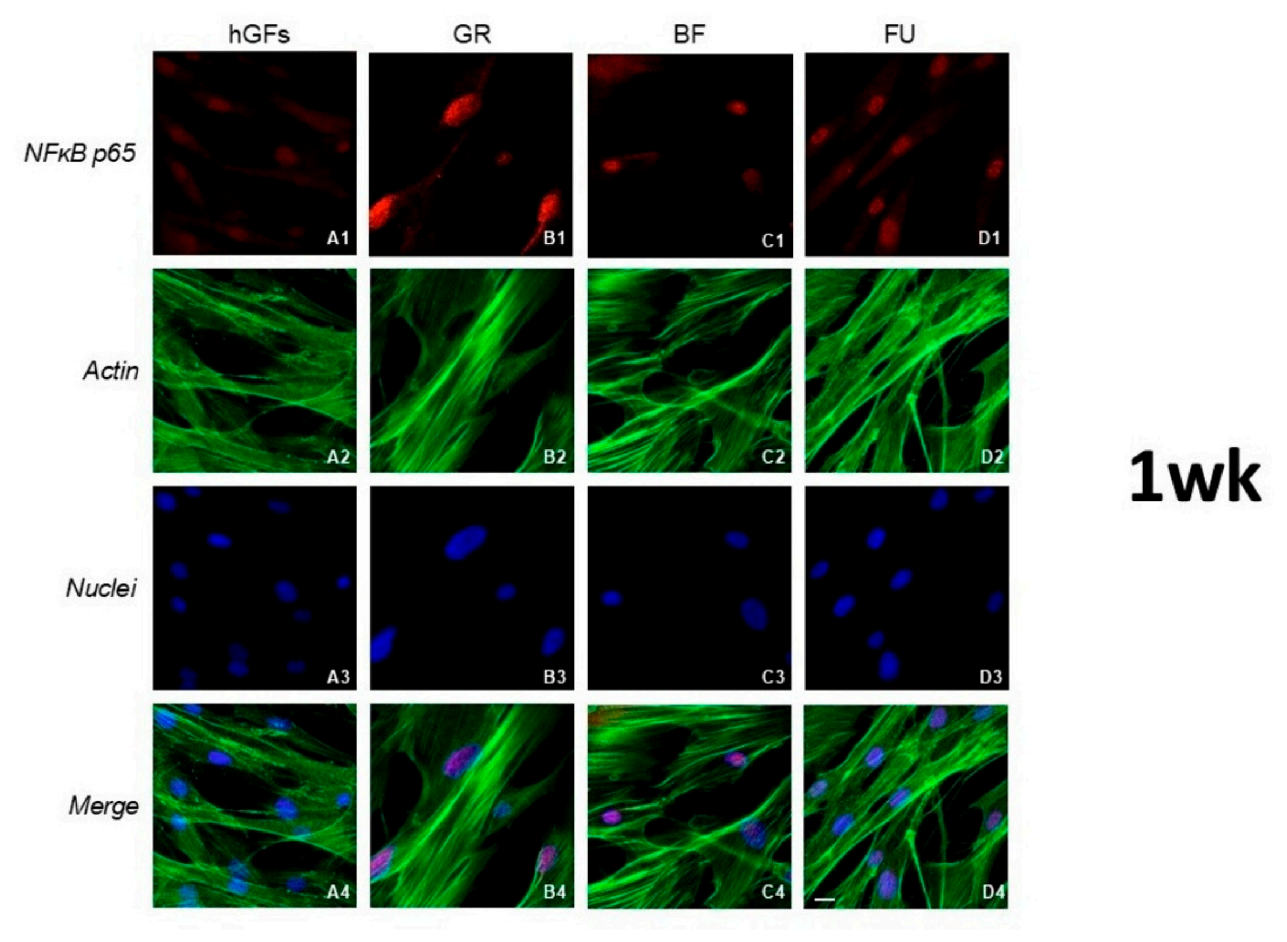
Immunofluorescence detection of NFkB p65 in hGFs (**A1**) and after 1 week exposure to GR (**B1**), BF (**C1**) and FU (**D1**) (red channel); actin image of all channels in the CTRL group (**A2**) and after 1-week exposure to GR (**B2**), BF (**C2**) and FU (**D2**); nuclei images of all channels in the CTRL group (**A3**) and after 1-week exposure to GR (**B3**), BF (**C3**) and FU (**D3**). Merged image of all channels (**A4**) and 1-week exposure to GR (**B4**), BF (**C4**) and FU (**D4**). Green fluorescence: cytoskeleton actin. Red fluorescence: NFkB p65. Blue fluorescence: cell nuclei. Mag: 40×. Scale bar: 10 µm. The purple color in the merged images results from the overlap of the red marker and the blue nuclear fluorescence.

**Figure 6:**
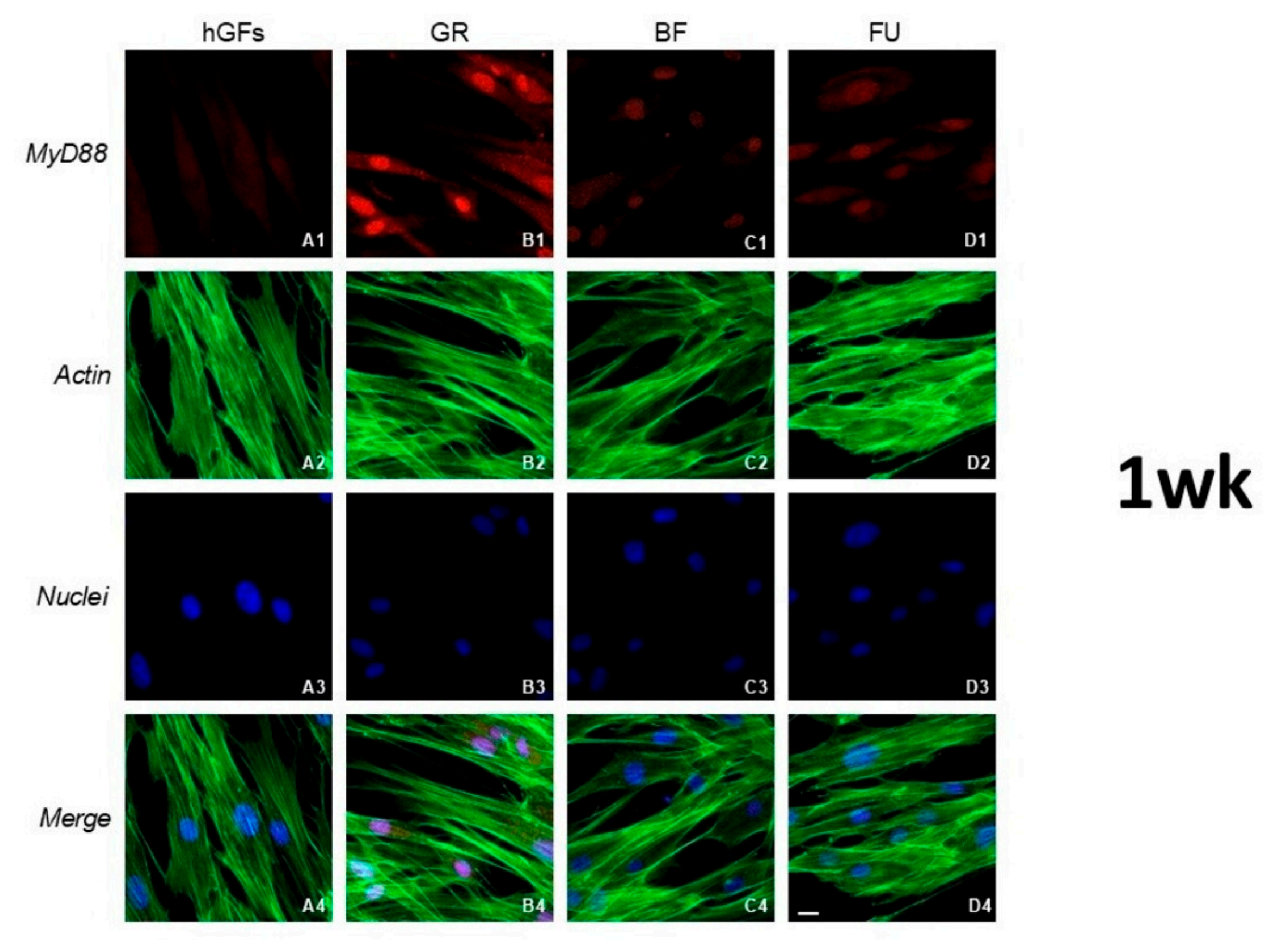
Immunofluorescence detection of MyD88 in hGFs (**A1**) and after 1-week exposure to GR (**B1**), BF (**C1**) and FU (**D1**) (red channel); actin image of all channels in the CTRL group (**A2**) and after 1-week exposure to GR (**B2**), BF (**C2**) and FU (**D2**); nuclei images of all channels in the CTRL group (**A3**) and after 1-week-exposure to GR (**B3**), BF (**C3**) and FU (**D3**). Merged image of all channels (**A4**) and 1-week exposure to GR (**B4**), BF (**C4**) and FU (**D4**). Green fluorescence: cytoskeleton actin. Red fluorescence: MyD88. Blue fluorescence: cell nuclei. Mag: 40×. Scale bar: 10 µm. The purple color in the merged images results from the overlap of the red marker and the blue nuclear fluorescence.

**Figure 7:**
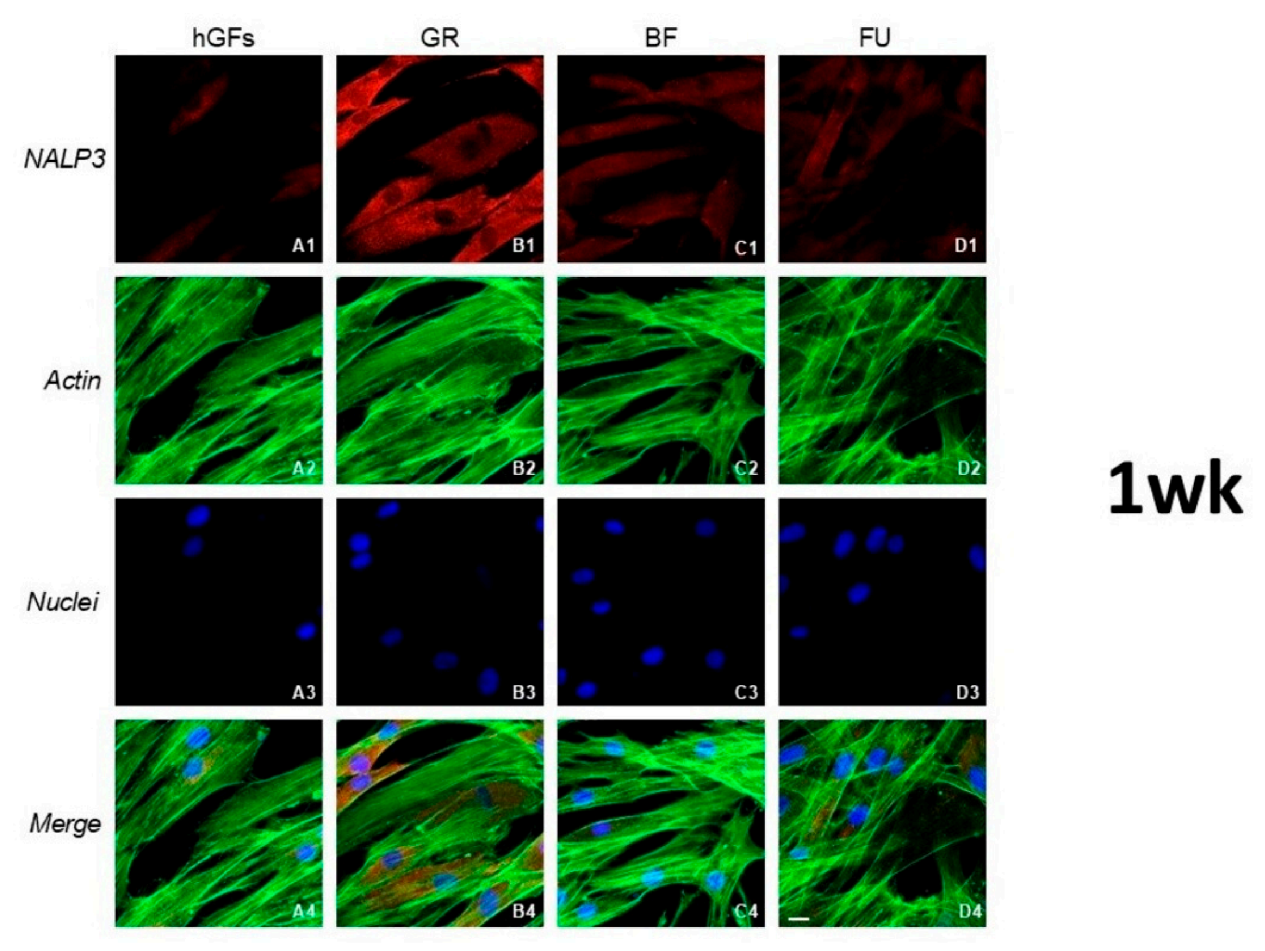
Immunofluorescence detection of NALP3 in hGFs (**A1**) and after 1-week exposure to GR (**B1**), BF (**C1**) and FU (**D1**) (red channel); actin image of all channels in the CTRL group (**A2**) and after 1-week exposure to GR (**B2**), BF (**C2**) and FU (**D2**); nuclei images of all channels in the CTRL group (**A3**) and after 1-week exposure to GR (**B3**), BF (**C3**) and FU (**D3**). Merged image of all channels (**A4**) and 1-week exposure to GR (**B4**), BF (**C4**) and FU (**D4**). Green fluorescence: cytoskeleton actin. Red fluorescence: NALP3. Blue fluorescence: cell nuclei. Mag: 40×. Scale bar: 10 µm.

**Figure 8:**
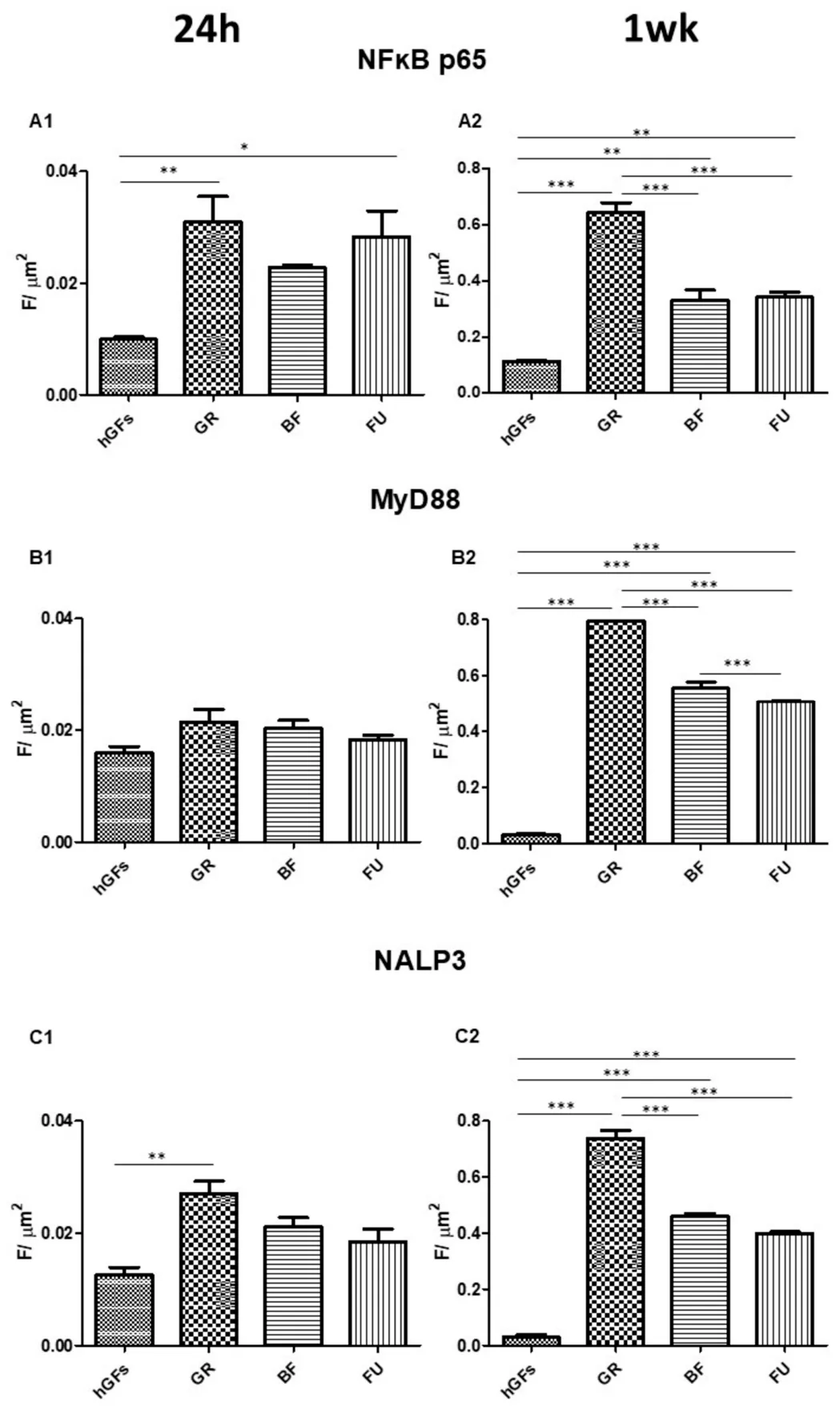
Quantification of expression of inflammatory markers after 24 h and 1 week of treatment. Quantitative analyses of NFkB p65 expression after 24 h (**A1**) and 1 week (**A2**); MyD88 expression after 24 h (**B1**) and 1 week (**B2**); NALP3 expression after 24 h (**C1**) and 1 week (**C2**). Immunofluorescence quantitative analysis expression was calculated as arbitrary unit of fluorescence per cell surface unit (F/μm^2^). Data are expressed as mean ± S.E.M. * *p* < 0.05; ** *p* < 0.01; *** *p* < 0.001.

**Figure 9:**
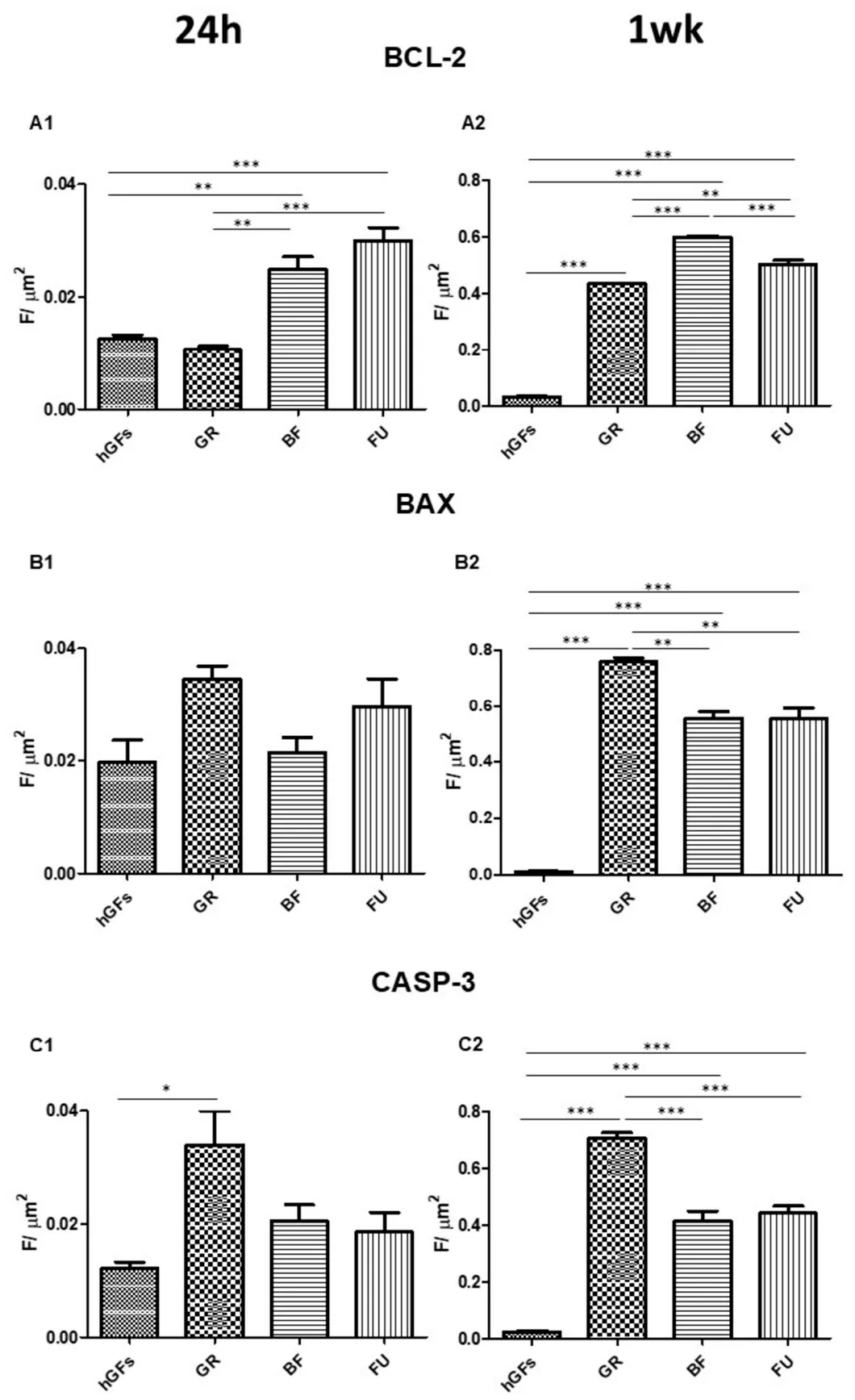
Quantification of expression of apoptotic markers after 24 h and 1 week of treatment. Quantitative analyses of BCL-2 expression after 24 h (**A1**) and 1 week (**A2**); BAX expression after 24 h (**B1**) and 1 week (**B2**); CASP-3 expression after 24 h (**C1**) and 1 week (**C2**). Immunofluorescence quantitative analysis expression was calculated as arbitrary unit of fluorescence per cell surface unit (F/μm^2^). Data are expressed as mean ± S.E.M. * *p* < 0.05; ** *p* < 0.01; *** *p* < 0.001.

**Figure 10:**
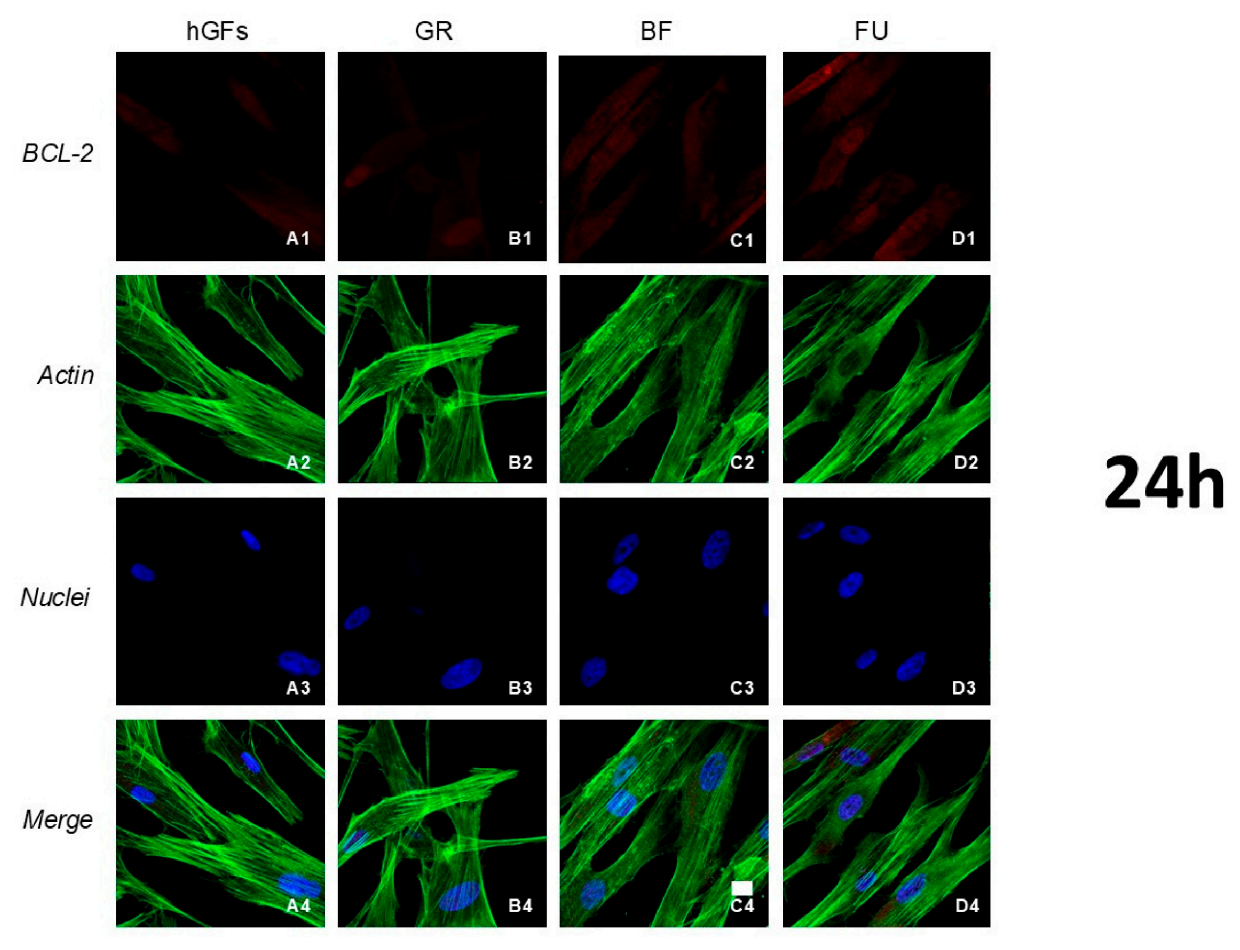
Immunofluorescence detection of BCL-2 in hGFs (**A1**) and after 24 h of exposure to GR (**B1**), BF (**C1**) and FU (**D1**) (red channel); actin images of all channels in the CTRL group (**A2**) and after 24 h exposure to GR (**B2**), BF (**C2**) and FU (**D2**); nuclei images of all channels in the CTRL group (**A3**) and after 24 h exposure to GR (**B3**), BF (**C3**) and FU (**D3**). Merged image of all channels (**A4**) and 24 h exposure to GR (**B4**), BF (**C4**) and FU (**D4**). Green fluorescence: cytoskeleton actin. Red fluorescence: BCL-2. Blue fluorescence: cell nuclei. Mag: 40×. Scale bar: 10 µm.

**Figure 11:**
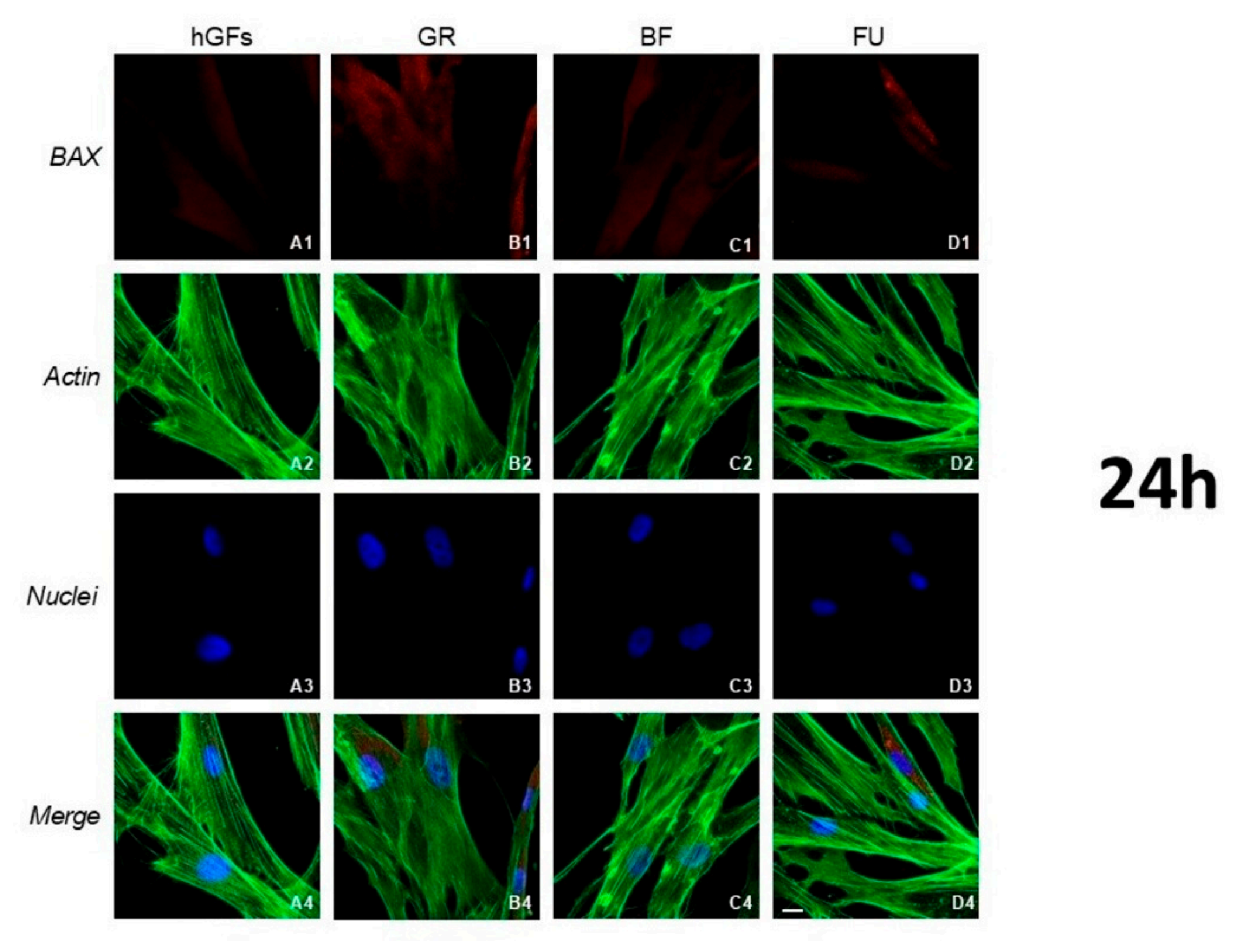
Immunofluorescence detection of BAX in hGFs (**A1**) and after 24 h of exposure to GR (**B1**), BF (**C1**) and FU (**D1**) (red channel); actin image of all channels in the CTRL group (**A2**) and after 24 h exposure to GR (**B2**), BF (**C2**) and FU (**D2**); nuclei images of all channels in the CTRL group (**A3**) and after 24 h exposure to GR (**B3**), BF (**C3**) and FU (**D3**). Merged image of all channels (**A4**) and 24 h exposure to GR (**B4**), BF (**C4**) and FU (**D4**). Green fluorescence: cytoskeleton actin. Red fluorescence: BAX. Blue fluorescence: cell nuclei. Mag: 40×. Scale bar: 10 µm.

**Figure 12:**
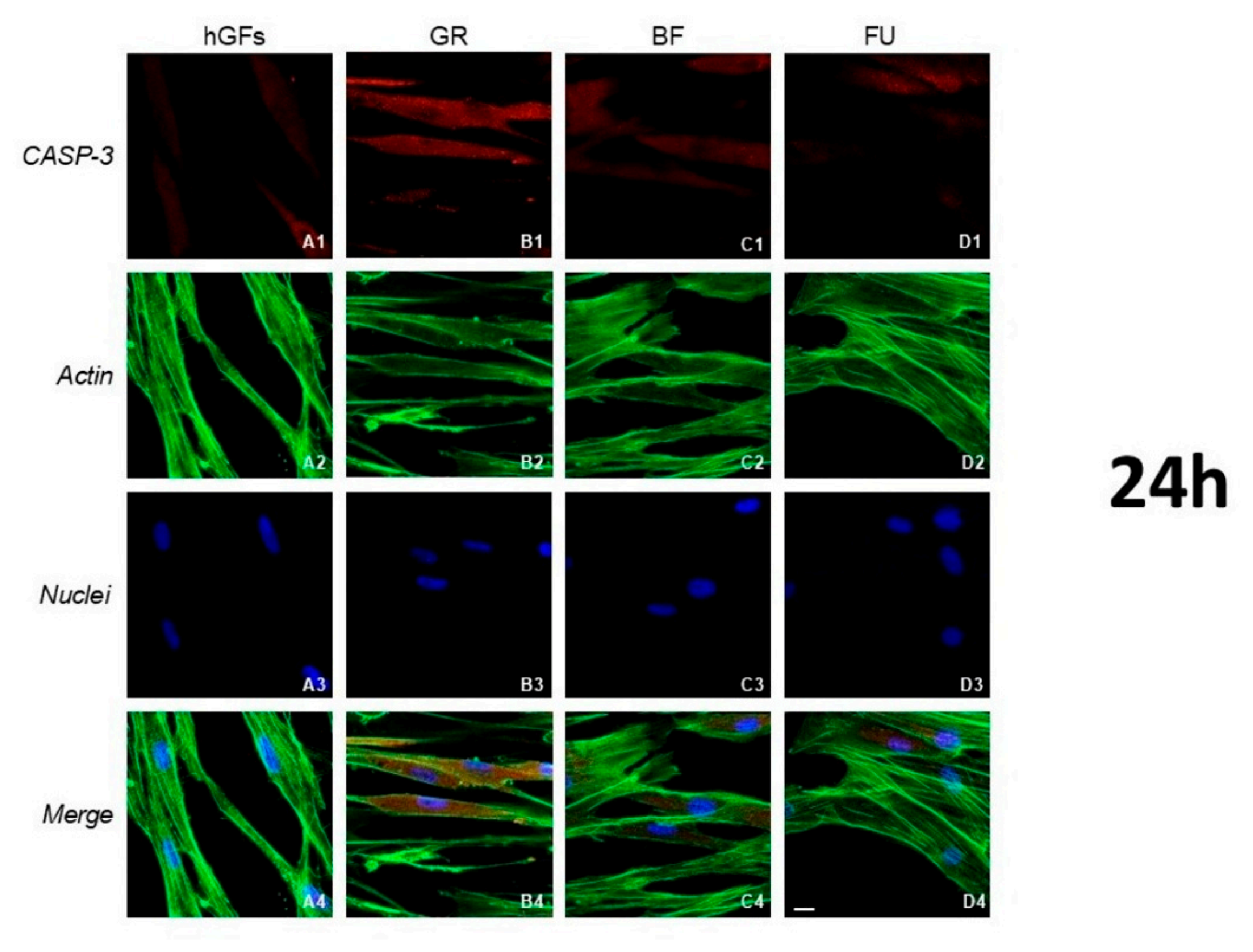
Immunofluorescence detection of CASP-3 in hGFs (**A1**) and after 24 h of exposure to GR (**B1**), BF (**C1**) and FU (**D1**) (red channel); actin image of all channels in the CTRL group (**A2**) and after 24 h exposure to GR (**B2**), BF (**C2**) and FU (**D2**); nuclei images of all channels in the CTRL group (**A3**) and after 24 h exposure to GR (**B3**), BF (**C3**) and FU (**D3**). Merged image of all channels (**A4**) and 24 h exposure to GR (**B4**), BF (**C4**) and FU (**D4**). Green fluorescence: cytoskeleton actin. Red fluorescence: CASP-3. Blue fluorescence: cell nuclei. Mag: 40×. Scale bar: 10 µm.

**Figure 13:**
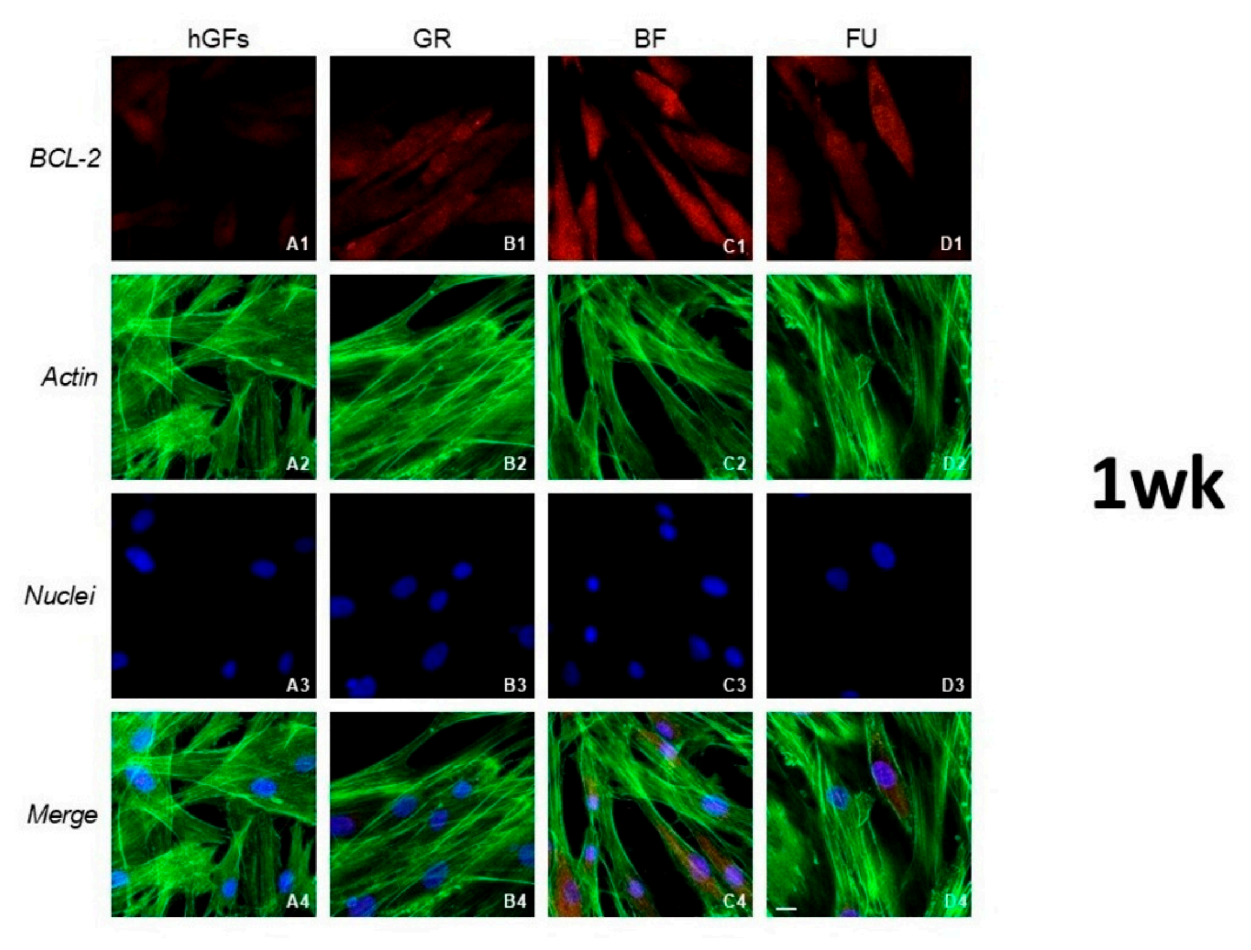
Immunofluorescence detection of BCL-2 in hGFs (**A1**) and after 1-week exposure to GR (**B1**), BF (**C1**) and FU (**D1**) (red channel); actin image of all channels in the CTRL group (**A2**) and after 1-week exposure to GR (**B2**), BF (**C2**) and FU (**D2**); nuclei images of all channels in the CTRL group (**A3**) and after 1-week exposure to GR (**B3**), BF (**C3**) and FU (**D3**). Merged image of all channels (**A4**) and 1-week exposure to GR (**B4**), BF (**C4**) and FU (**D4**). Green fluorescence: cytoskeleton actin. Red fluorescence: BCL-2. Blue fluorescence: cell nuclei. Mag: 40×. Scale bar: 10 µm.

**Figure 14:**
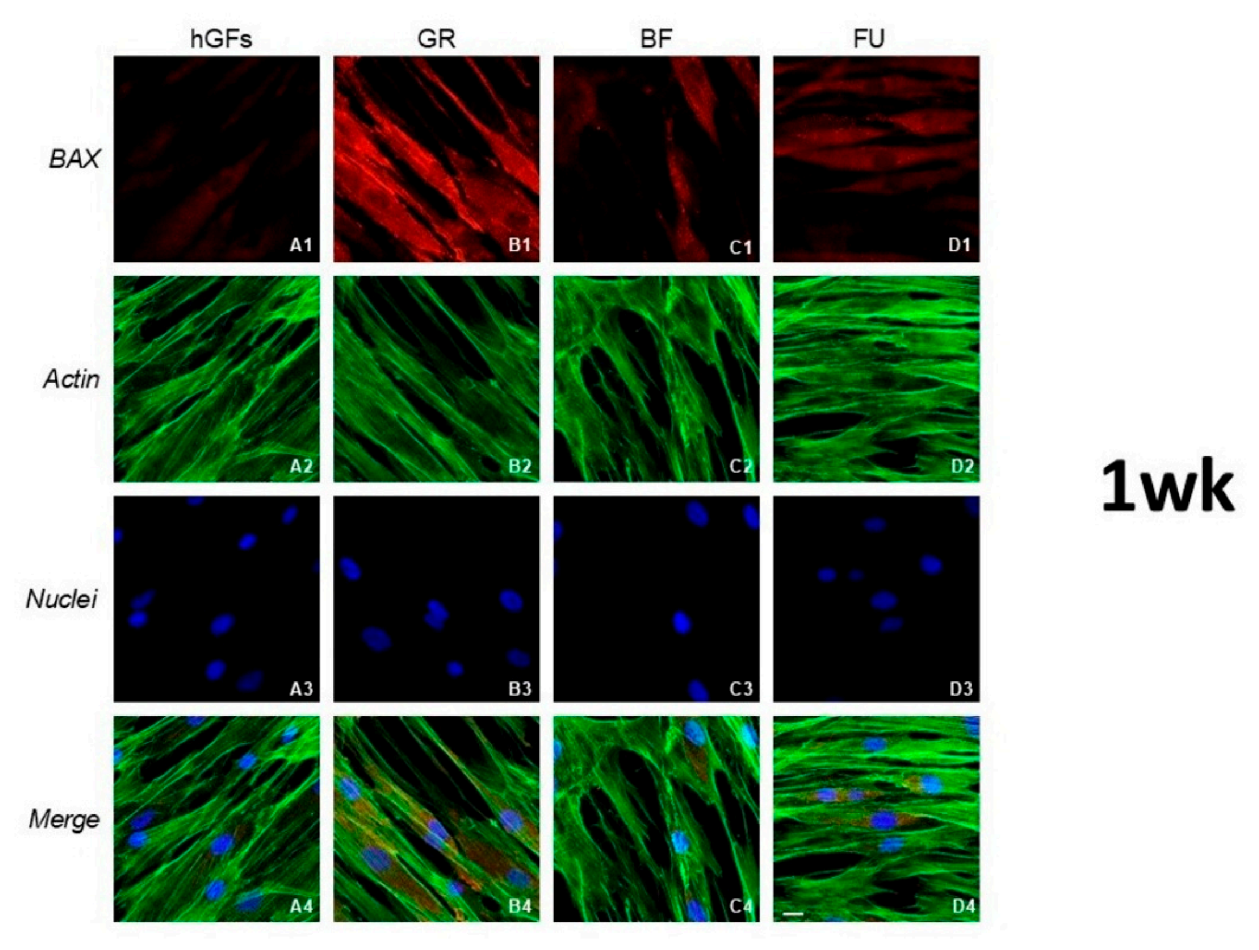
Immunofluorescence detection of BAX in hGFs (**A1**) and after 1-week exposure to GR (**B1**), BF (**C1**) and FU (**D1**) (red channel); actin image of all channels in the CTRL group (**A2**) and after 1-week exposure to GR (**B2**), BF (**C2**) and FU (**D2**); nuclei images of all channels in the CTRL group (**A3**) and after 1-week exposure to GR (**B3**), BF (**C3**) and FU (**D3**). Merged image of all channels (**A4**) and 1-week exposure to GR (**B4**), BF (**C4**) and FU (**D4**). Green fluorescence: cytoskeleton actin. Red fluorescence: BAX. Blue fluorescence: cell nuclei. Mag: 40×. Scale bar: 10 µm.

**Figure 15:**
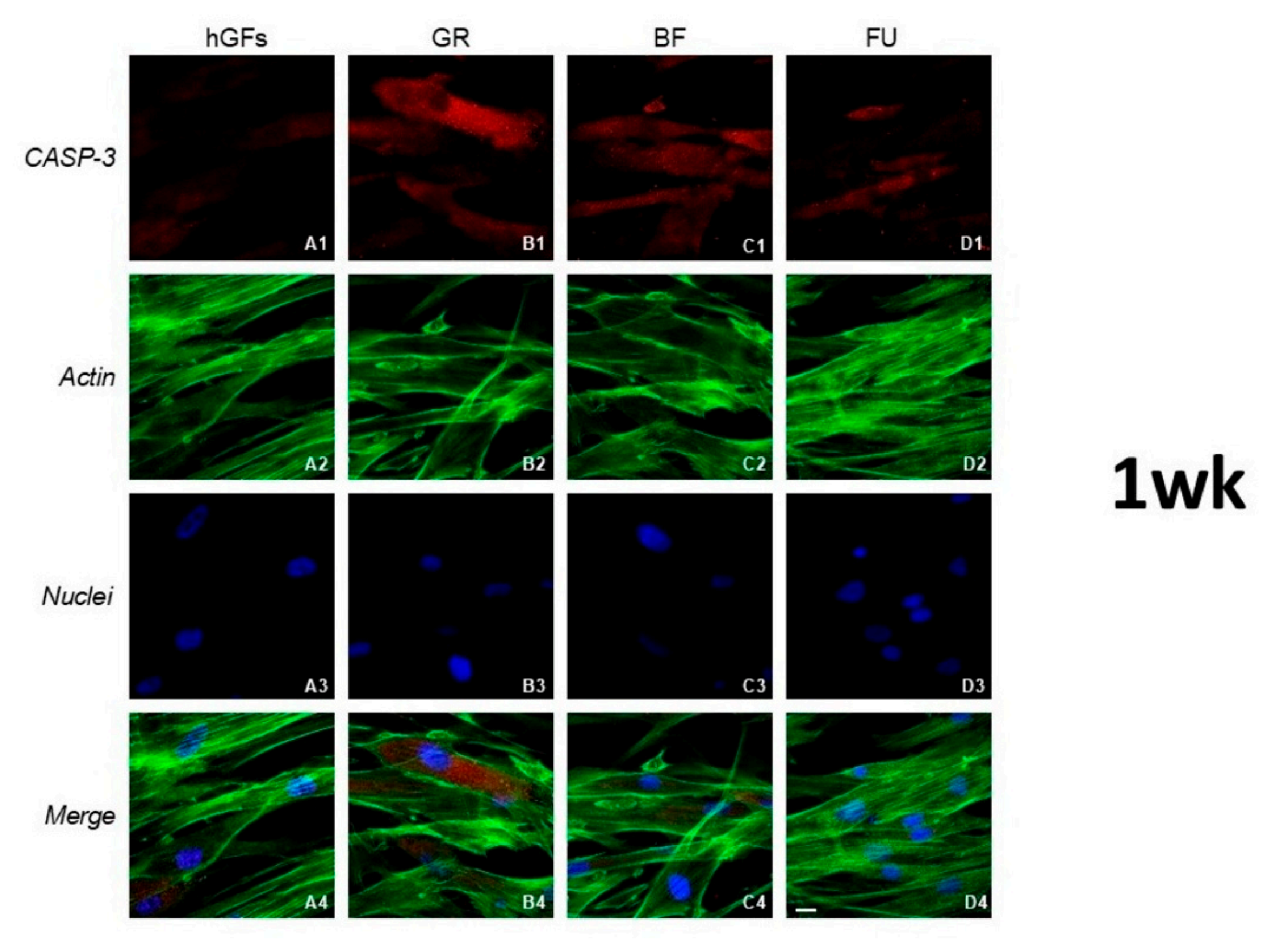
Immunofluorescence detection of CASP-3 in hGFs (**A1**) and after 1-week exposure to GR (**B1**), BF (**C1**) and FU (**D1**) (red channel); actin image of all channels in the CTRL group (**A2**) and after 1-week exposure to GR (**B2**), BF (**C2**) and FU (**D2**); nuclei images of all channels in the CTRL group (**A3**) and after 1-week exposure to GR (**B3**), BF (**C3**) and FU (**D3**). Merged image of all channels (**A4**) and 1-week exposure to GR (**B4**), BF (**C4**) and FU (**D4**). Green fluorescence: cytoskeleton actin. Red fluorescence: CASP-3. Blue fluorescence: cell nuclei. Mag: 40×. Scale bar: 10 µm.

**Figure 16:**
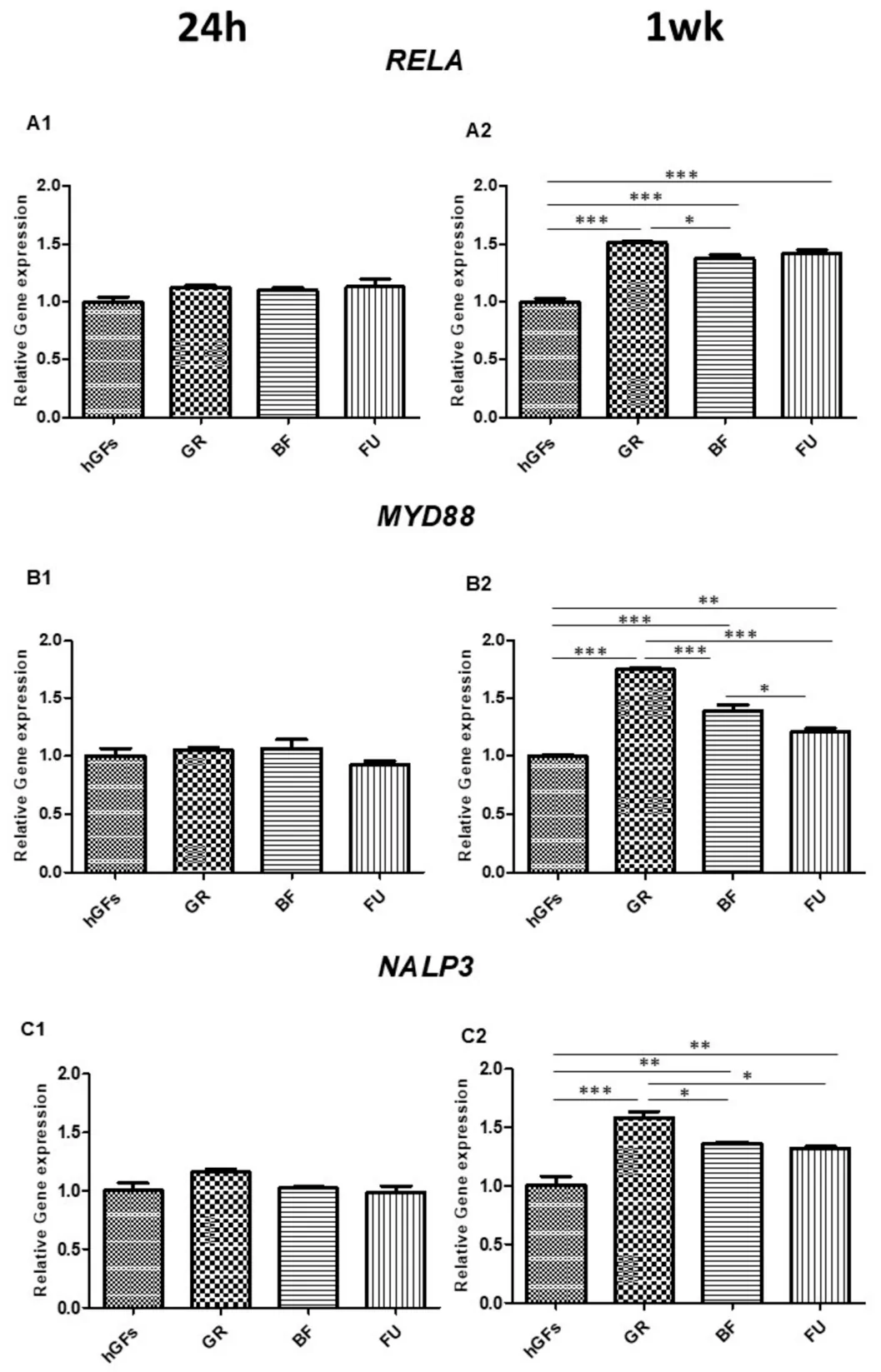
*RELA*, *MYD88*, and *NALP3* gene expression. (**A1**,**B1**,**C1**) Histograms of real-time PCR for *RELA*-, *MYD88*-, and *NALP3*-related gene expression in untreated cells (CTRL) and GR, BF, and FU samples after 24 h of exposure. (**A2**,**B2**,**C2**) Histograms of real-time PCR for *RELA*-, *MYD88*-, and *NALP3*-related gene expression in untreated cells (CTRL) and GR, BF, and FU samples after 1-week exposure. * *p* < 0.05; ** *p* < 0.01; *** *p* < 0.001.

**Figure 17:**
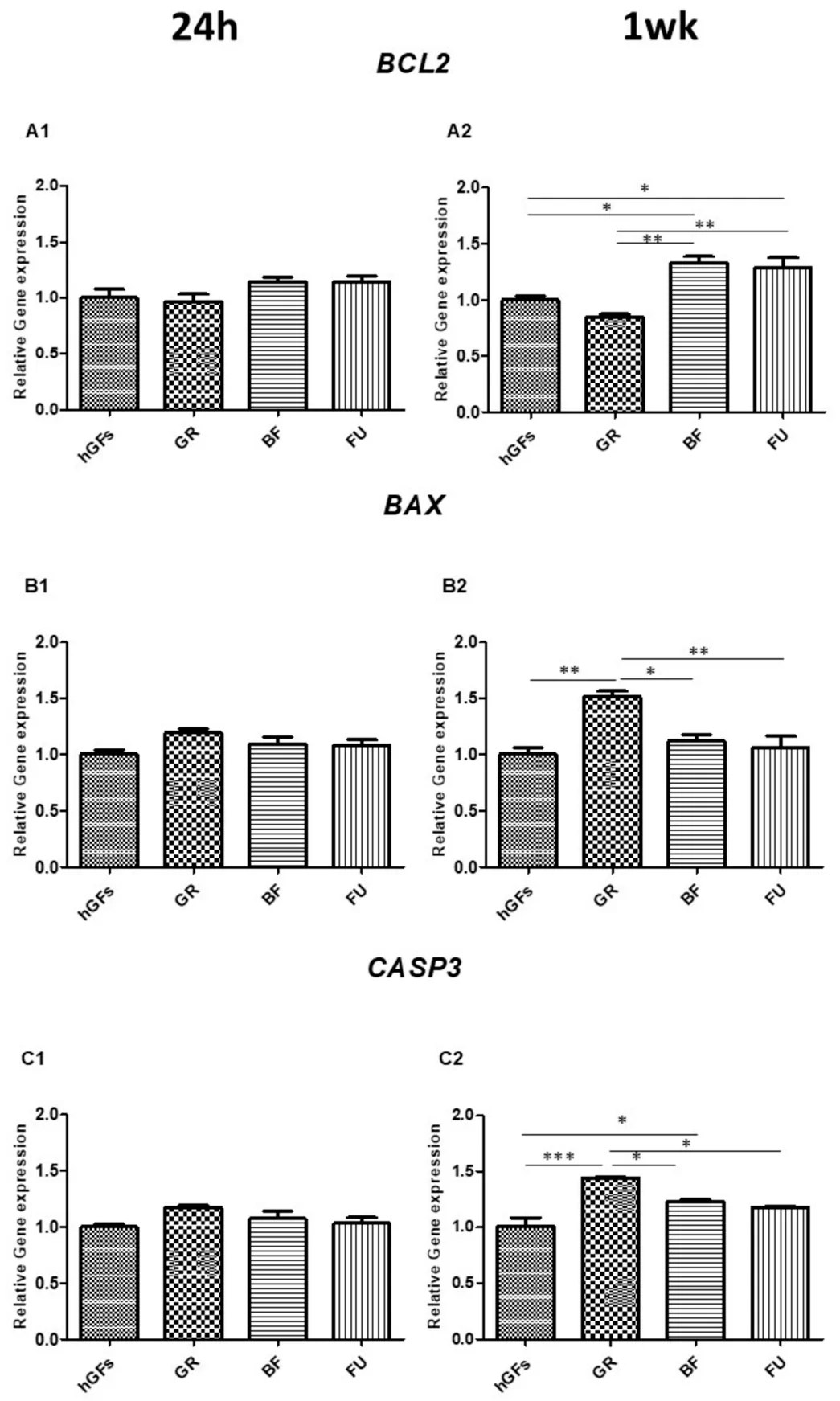
*BCL2*, *BAX*, and *CASP3* gene expression. (**A1**,**B1**,**C1**) Histograms of real-time PCR for *BCL2*-, *BAX*- and *CASP3*-related gene expression in untreated cells (CTRL) and GR, BF, and FU samples after 24 h of exposure. (**A2**,**B2**,**C2**) Histograms of real-time PCR *BCL2*-, *BAX*- and *CASP3*-related gene expression in untreated cells (CTRL) and GR, BF, and FU samples after 1-week exposure. * *p* < 0.05; ** *p* < 0.01; *** *p* < 0.001.

**Figure 18:**
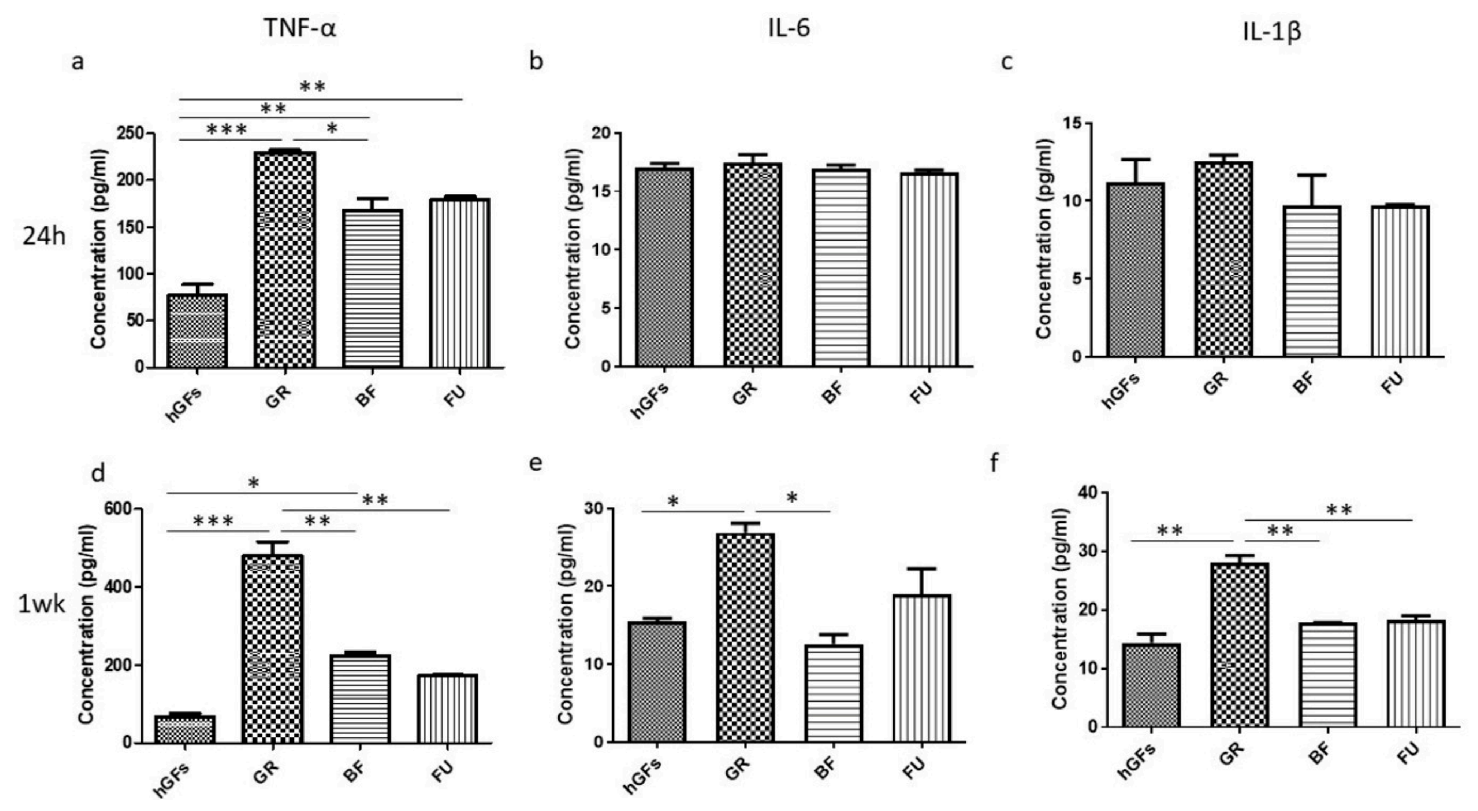
ELISA assay. Inflammatory effects of GR, BF and FU on hGFs. Levels of proinflammatory cytokines TNF-α (**a**,**d**), IL-6 (**b**,**e**), and IL-1β (**c**,**f**) released after 24 h and 1 week of incubation with GR, BF and FU. Cytokine concentrations were measured using ELISA assay. Data are presented as mean ± SEM. (* *p* < 0.05, ** *p* < 0.01, *** *p* < 0.001).

**Table 1:** Composition of the experimental groups is summarized in the following table.

Experimental Group	Material	Manufacturer	Batch	Composition
GR	GrandioSO —Shade A2	Voco GmbH (Cuxhaven, Germany)	2213772	89% (*w*/*w*) fillers (1 μm glass ceramic filler, 20 nm–40 nm silicon dioxide fillers), Bis-GMA, Bis-EMA, Teg-DMA.
BF	Enamel Plus HRi Biofunction —Shade BF2	Micerium S.p.A. (Avegno, Genova, Italy)	2024006140	74% in weight (60% in volume) fillers (0.005 μm–0.05 μm silicon dioxide fillers), (0.2–3.0 μm glass fillers), Urethane dimethacrylate, Tricyclodecane dimethanol dimethacrylate.
FU	3M™ Filtek™ Universal Restorative—Shade A2	3M ESPE (St Paul, MN, USA)	10544071	Fillers: combination of a non-agglomerated 20 nm silica filler, non-agglomerated 4 to 11 nm zirconia filler, ytterbium trifluoride filler made of agglomerated 100 nm particles (76.5% *w*/*w*), Aromatic Urethane Dimethacrylate, Diurethane Dimethacrylate, 1,12-Dodecane Dimethycrylate.

**Table 2:** Primer sequences used for real-time PCR reactions.

Gene	Primer 1	Primer 2
	Sequence (5′-3′)	Sequence (5′-3′)
RELA	5′-CGAGCTTGTAGGAAAGGACTG-3′	5′-TGACTGATAGC-CTGCTCCAG-3′
MYD88	5′-CGGTCTCCTCCACATCCT-3′	5′-GCCGGATCTCCAAGTACTCA’-3′
NALP3	5′-GAATGCCTTGG GAGACTCAG-3′	5′-AGATTCTGATT AGTGCTGAGTACC-3′
BCL2	5′-GATGACTGAGTACCTGAACCG 3′	5′-ACCAATCTTGT AGGACTGACC-3′
BAX	5′-TCTGAGCAGATCATGAAGACAG-3′	5′-AGCCAGGAGAAATCAAACAGAG- 3′
CASP3	5′-CTCTGGAATATCCCTGGACAAC-3′	5′GTTTGCTGCATCGACATCTG-3′
B2M	5′-GGACTGGTCTT-TCTATCTCTTGT-3′	5′-ACCTCCATGATGCTGCTTAC-3′

## Data Availability

The original contributions presented in this study are included in the article. Further inquiries can be directed to the corresponding author.
